# Preparation of glass-ionomer cement containing ethanolic Brazilian pepper extract (*Schinus terebinthifolius* Raddi) fruits: chemical and biological assays

**DOI:** 10.1038/s41598-020-79257-3

**Published:** 2020-12-18

**Authors:** Isabelle C. Pinto, Janaína B. Seibert, Luciano S. Pinto, Vagner R. Santos, Rafaela F. de Sousa, Lucas R. D. Sousa, Tatiane R. Amparo, Viviane M. R. dos Santos, Andrea M. do Nascimento, Gustavo Henrique Bianco de Souza, Walisson A. Vasconcellos, Paula M. A. Vieira, Ângela L. Andrade

**Affiliations:** 1 Departamento de Química, Universidade Federal de Ouro Preto, UFOP, Ouro Preto, 35400-000 Brazil; 2 Departamento de Química, Universidade Federal de São Carlos, UFSCar, São Carlos, 13565-905 Brazil; 3 Departamento de Clínica, Patologia e Cirurgias Odontológicas, Universidade Federal de Minas Gerais, UFMG, Belo Horizonte, 31270-901 Brazil; 4 Departamento de Ciências Biológicas, Universidade Federal de Ouro Preto, UFOP, Ouro Preto, 35400-000 Brazil; 5 Laboratório de Fitotecnologia, Universidade Federal de Ouro Preto, UFOP, Ouro Preto, 35400-000 Brazil; 6 Departamento de Odontologia Restauradora, Universidade Federal de Minas Gerais, UFMG, Belo Horizonte, 31270-901 Brazil

**Keywords:** Biological techniques, Biotechnology, Plant sciences, Health care, Medical research

## Abstract

Plants may contain beneficial or potentially dangerous substances to humans. This study aimed to prepare and evaluate a new drug delivery system based on a glass-ionomer-Brazilian pepper extract composite, to check for its activity against pathogenic microorganisms of the oral cavity, along with its in vitro biocompatibility. The ethanolic Brazilian pepper extract (BPE), the glass-ionomer cement (GIC) and the composite GIC-BPE were characterized by scanning electron microscopy, attenuated total reflectance Fourier transform infrared spectroscopy (ATR-FTIR), and thermal analysis. The BPE compounds were identified by UPLC–QTOF–MS/MS. The release profile of flavonoids and the mechanical properties of the GIC-BPE composite were assessed. The flavonoids were released through a linear mechanism governing the diffusion for the first 48 h, as evidenced by the M_t_/M_∞_ relatively to $\sqrt t$, at a diffusion coefficient of 1.406 × 10^–6 ^cm^2^ s^−1^. The ATR-FTIR analysis indicated that a chemical bond between the GIC and BPE components may have occurred, but the compressive strength of GIC-BPE does not differ significantly from that of this glass-ionomer. The GIC-BPE sample revealed an ample bacterial activity at non-cytotoxic concentrations for the human fibroblast MRC-5 cells. These results suggest that the prepared composite may represent an alternative agent for endodontic treatment.

## Introduction

It is established that the bacterial flora of the gingival crevice may help better understand the etiology of periodontal disease^1^ and, thus, orient its treatment by controlling this microflora. Although systemic administration of conventional antibiotics has been proved to be a useful method of controlling such a sub-gingival infection^2^, the challenge now turns to be preventing the indiscriminate antibiotic therapy, which may very likely lead to systemic side-effects, appearance of resistant strains, and superimposed infections. This idea strongly supports the search of clinically more efficient alternative treatments, with no significant collateral effect to the patient: it is envisaged to develop a suitably designed mini-support to carry and locally deliver the pharmacological principle at a specific point in the buccal cavity. The supporting material anchoring the active principle must be biologically safe and allows delivering the therapeutic drug at a rate assuring its optimum anti-biotic action.

The use of phytotherapic principles to treat, cure and prevent human diseases is one of the oldest forms of medicinal practice of the humankind. Nevertheless, since the appearance of synthetic drugs, starting by the aspirin, in 1897, there has been a decreasing interest in plant-derived therapeutics^3^. Herbal medicine was seen as an “unqualified, non-scientific and primitive medicinal practice”, used by people without access to the “real” medicine^3^. However, in more recent years phytotherapeutics have become again popular, which can be seen by the number of scientific papers published on this subject. Nevertheless, some plants have the potential to be poisoning to humans. Plants produce a plethora of metabolic compounds acting to self-protect them from invasion by microorganisms and viruses as well as from the herbivorous macro-fauna^4^. These complex substances include several classes of compounds, some of which are lethal to humans even if consumed in minute quantities^4^. Because of this potential toxicity, it is always important to perform biocompatibility testing when trying the use of a given plant extract against microorganisms.

*Schinus terebinthifolius* Raddi, from the Anacardiaceae family, is a medicinal plant, native of the Brazilian flora, and is commonly known as Brazilian pepper or Aroeira. Previous studies have shown that *S. terebinthifolius* does contain phenolic compounds with different biological activities, including antimicrobial, antiulcerogenic, anticancer, antihistamine, antihypertensive, antihyperalgesic, wound healing, anti-inflammatory and antioxidant activities in different models^5–13^.

Despite of the various studies of Brazilian pepper extract (BPE) in dentistry, there are very few studies dealing with the ethanolic extract of Brazilian pepper fruit incorporated into GICs and no study at all on the releasing profile of the flavonoids of the pepper extract, incorporated into the ionomer, has been reported. Taking into regard that the drug release profile is critical to allow proposing changes in the synthesis protocol towards its better clinical use^14^, the purpose of this work was to add the BPE to the GIC, in an effort to know more in deep about the enhanced antibacterial activity mainly due to the flavonoids and to attempt developing a new therapeutic alternative agent, which may be used in endodontic treatments.

Being the focus of this work the development of a phytotherapic anti-biotic carrier, the glass-ionomer cement (GIC), a material basically consisting of a basic glass and acidic polymer, as previously proposed by Wilson and Kent^15^, was now studied in more details. The glass-ionomer was chosen for its ability, from among many of its potential uses, to act as drug delivery supporting systems^16^.

## Results

### Characterization of the samples

**Figure 1:**
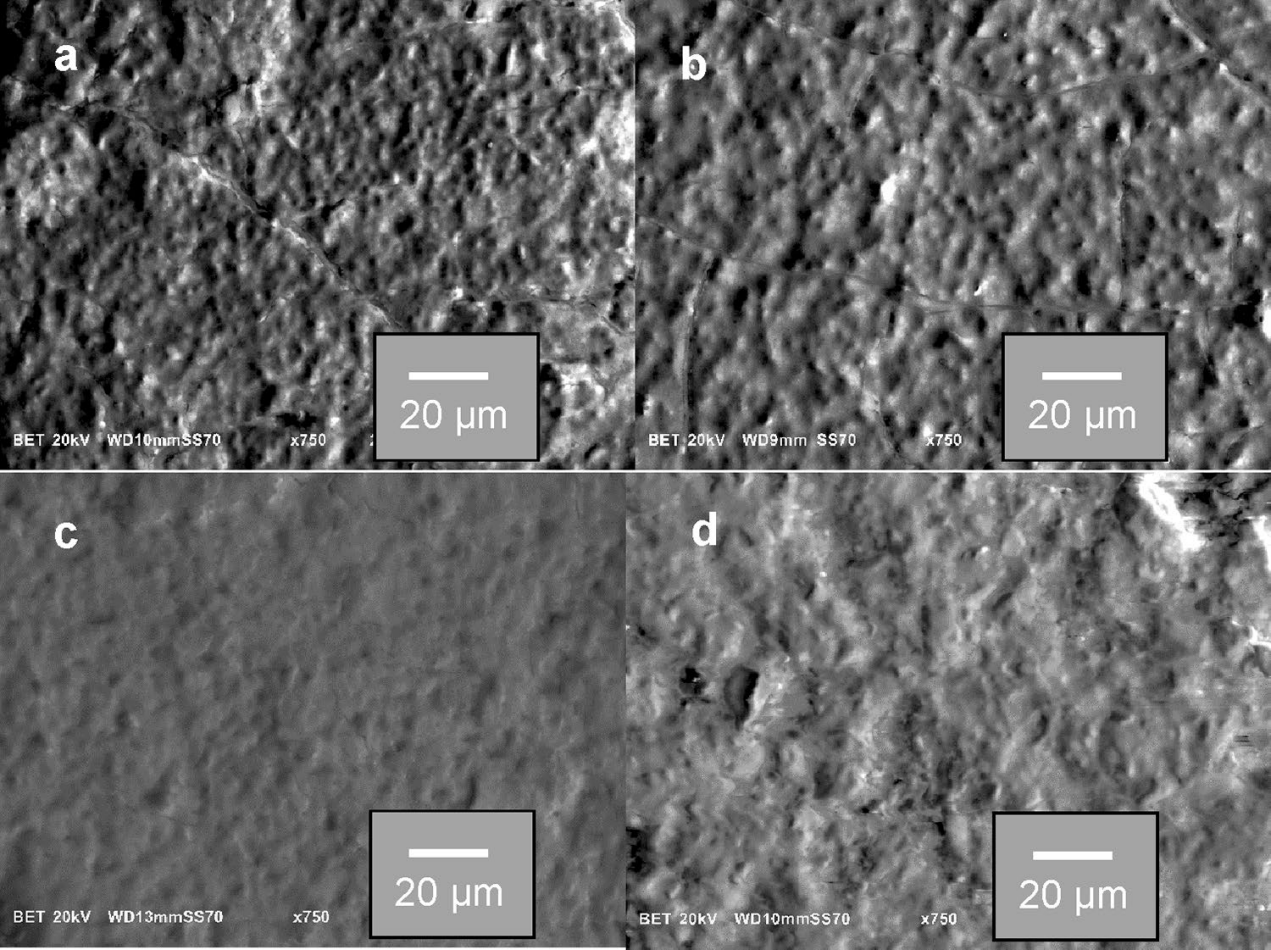
Microphotographs of glass-ionomer cements samples: (**a**) GIC and (**b**) GIC-BPE, before immersion in SBF solution; and (**c**) GIC and (**d**) GIC-BPE, after immersion in SBF solution for 96 h.

Figure 1 shows the SEM image of GIC and GIC-BPE samples, before (Fig. 1a,b, respectively) and after (Fig. 1c,d, respectively) immersion in SBF. There were significant differences in the initial surface roughness of the samples before and after immersion in SBF. After drug loading, the samples were apparently smoother.

**Figure 2:**
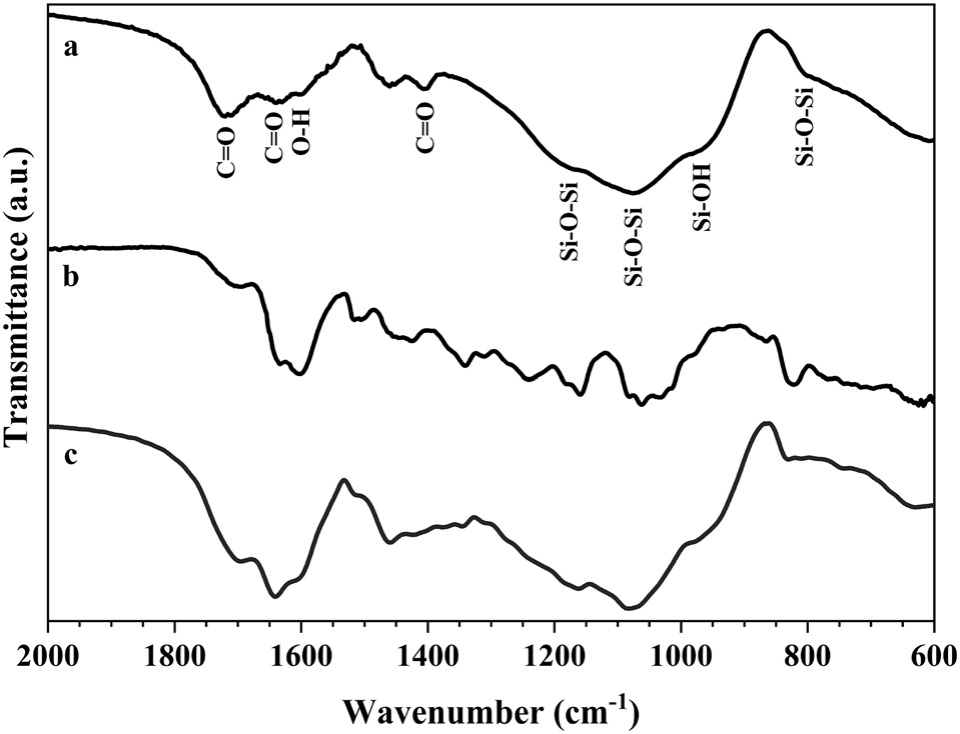
ATR-FTIR spectra of: (**a**) GIC, (**b**) BPE, and (**c**) GIC-BPE.

Figure 2 shows the ATR-FTIR absorption spectra of GIC, BPE and GIC-BPE samples. The characteristic peaks are listed in the Table 1.

**Table 1:** The preliminary assignments of ATR-FTIR absorption spectra of GIC, BPE and GIC-BPE samples.

Sample	Bands (cm^−1^)	Preliminary assignments	Main attribution	References
GIC sample (Fig. 2a)	1700 and 1650	C=O stretching	Carbonyl group	^17^
	1604	OH deformation	Aromatic ring vibration	^17^
	1402	C=O stretching	Carboxylate for surface hydroxyl group	^18^
	1170, 1080 and 800	Si–O–Si asymmetric stretching and Si–O–Si bending	Siloxane	^18–20^
	950	Si–OH stretching	Silanol	^18–20^
BPE sample^a^ (Fig. 2b)	1680	C=O stretching	Phenolic compounds and flavonoids	^21^
	1150–1050 cm^−1^ and 900–1300 cm^−1^	C–OH and CH groups, respectively	Phenolic compounds and flavonoids	^21^

^a^The broad absorption bands obtained from *Schinus terebinthifolius* Raddi represent the substantial overlap of absorption bands of various components with different contents.

**Figure 3:**
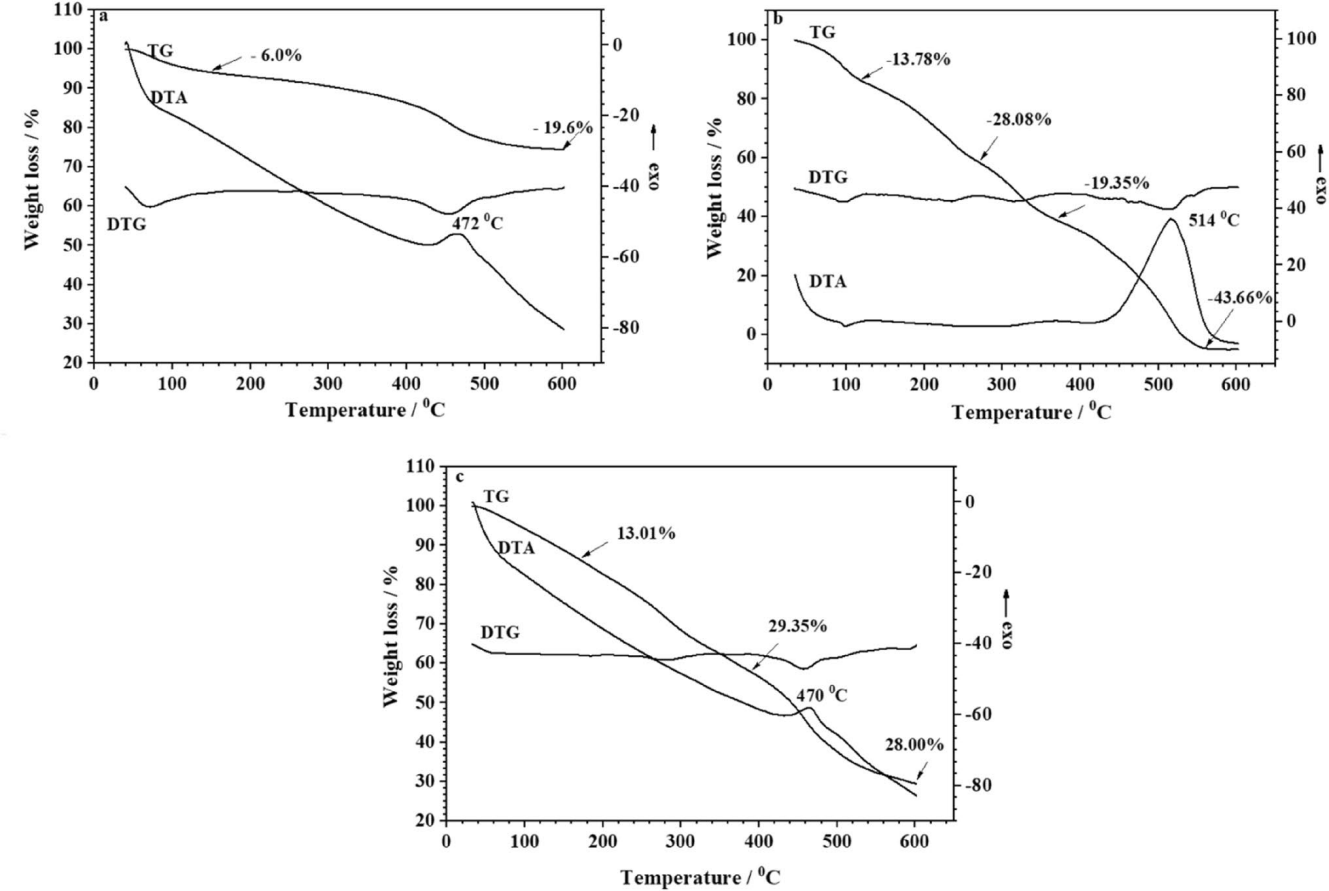
TG/DTG/DTA diagrams of samples: (**a**) GIC, (**b**) BPE, (**c**) GIC-BPE, before immersion in SBF.

The weight loss (TG) and derivative weight loss (DTG) curves of the GIC, BPE and GIC-BPE samples under an air environment are illustrated in Fig. 3. The TG for the GIC sample (Fig. 3a) shows two main stages of weight loss at the temperature intervals of 30–150 °C and 330–580 °C. The total loss up to 600 °C amounted to about 25.6% of the initial mass.

The pyrolysis process of *S. terebinthifolius* dry extract (BPE sample) showed four distinct mass loss zones (Fig. 3b).

The GIC-BPE specimen (Fig. 3c) presented weight losses at the temperature intervals of 30–270 °C, 270–391 °C and 391–600 °C (26.61%, 29.35% and 28.00%, respectively).

The DTA curve for the GIC sample (Fig. 3a) shows the presence of an exothermic peak at 472 °C. The DTA curve for the BPE sample (Fig. 3b) initially indicate an endothermic peak. After this, it shows an exothermic event. The DTA curve for the GIC-BPE sample (Fig. 3c) shows the presence of an exothermic peak at 470 °C.

To our knowledge, there is no study investigating the mechanical properties of the GIC-BPE composite. Our study shows that the incorporation of about 38 wt% BPE decreased the compressive strength of the GIC based composite from 176.60 ± 6.46 MPa to 161.17 ± 5.01 MPa in the composite GIC-BPE.

Phytochemical analyses revealed that alkaloid, anthraquinone, carbohydrate, and saponin were absent. The test was based on the colour changes after the reaction of the extract with standard reagents. The BPE sample presented a positive result for flavonoids, tannin and terpenoids.

Results of analyses performed on ethanolic BPE show that the total phenolic content was 60.09 ± 7.65 mg of galic acid/g of sample and the total flavonoids content was 1.88 ± 1.01 mg of quercetin/g of sample. Uliana et al.^13^ found a similar result for essential oil extracted from Brazilian pepper.

The chemical profile of BPE was performed using UPLC–QTOF–MS/MS analysis and the molecular formula of precursor ions (MS1 spectra) were calculated (accurate 3 ppm). Then, ions-fragment analysis (MS2 spectra) and proposed fragmentation mechanism were performed using molecular annotation from different metabolite databases (FooDB, PlantCyc, ChEBI, LipidMAPS, DrugBank, KNApSAcK, NANPDB, PubChem, UNPD, and METLIN) and confidence literature. Chromatogram of the BPE (Fig. S1) and the MS1 and MS2 data for each compound are described in the supplementary material (Figs. S2–S59). According to Table 2, 27 compounds were suggested, and their chemical structures are represented in the Fig. 4.

**Table 2:** Detected compounds from ethanolic extract of *Schinus terebinthifolius* (Brazilian pepper) by UPLC–QTOF–MS/MS.

No.	RT (min)	[M−H]^−^ (*m*/*z*)	Formula	Error (ppm)	Ions-fragment (*m*/*z*)	Annotation	Class	References
1	0.91	343.0667	C_14_H_16_O_10_	0.6	191.06; 169.01; 125.02; 93.04	Theogallin	GD	^22^
2	0.92	331.0668	C_13_H_16_O_10_	0.9	271.05; 241.04; 211.03; 169.01; 1250.02	Glucogallin	GD	^23^
3	3.05	325.0558	C_14_H_14_O_9_	− 0.6	185.02; 173.05; 169.01; 124.02; 111.05	5-Galloylshikimic acid	GD	^24^
4	3.26	495.0775	C_21_H_20_O_14_	0.0	343.07;325.06; 191.06; 173.05; 169.01; 125.02	3,4-Di-O-galloylquinic acid	GD	^25^
5	3.42	483.0773	C_20_H_20_O_14_	− 0.4	331.07; 271.05; 211.02; 169.01; 125.02	1,2-Digalloyl-β-D-glucopyranose	GD	^26^
6	4.30	321.0247	C_14_H_10_O_9_	0.0	169.01; 125.03	Digallic acid	GD	^27^
7	4.76	477.0669	C_21_H_18_O_13_	0.0	325.05; 307.04; 201.06; 169.01; 125.02	3,5-Di-O-galloylshikimic acid	GD	^24^
8	4.76	635.0882	C_27_H_24_O_18_	− 0.3	521.02; 483.08; 465.07; 331.07; 271.05; 169.01	1,2,6-Trigalloyl-β-D-glucopyranose	GD	^28^
9	5.39	473.0353	C_21_H_14_O_13_	− 0.6	321.03; 169.01; 125.02	Trigallic acid	GD	^29^
10	5.39	615.0986	C_28_H_24_O_16_	0.0	463.09; 301.03; 271.03	2′'-O-Galloylhyperin	GD/ FL	^30^
11	5.44	787.0987	C_34_H_28_O_22_	− 0.9	635.09; 617.08; 483.08; 465.07; 447.06; 403.05; 179.03	1,2,3,6-Tetragalloyl-β-D-glucopyranose	GD	^31^
12	5.47	169.0139	C_7_H_6_O_5_	1.2	125.02; 107.01; 97.03; 79.02	Gallic acid	GD	^32^
13	5.49	629.0778	C_28_H_22_O_17_	− 0.2	583.05; 477.07; 325.06; 169.01	Trigalloylshikimic acid	GD	^24^
14	5.65	463.0873	C_21_H_20_O_12_	− 0.9	317.03; 316.02; 271.02; 164.08	Myricitrin	FL	^33^
15	5.75	441.0821	C_22_H_18_O_10_	− 0.2	315.03; 289.07; 245.08; 137.03	Catechin 3-O-gallate	GD/CA	^34^
16	6.01	433.0774	C_20_H_18_O_11_	0.7	313.09; 301.04; 300.03	Avicularin	FL	^35^
17	6.17	447.0927	C_21_H_20_O_11_	0.0	300.03; 271.02; 255.03; 243.03; 151.00; 135.01	Quercitrin	FL	^36^
18	6.41	417.0819	C_20_H_18_O_10_	− 0.7	284.03; 255.03; 227.03	Kaempferol 3-O-α-L-arabinopyranoside	FL	^37^
19	6.80	287.0559	C_15_H_12_O_6_	1.0	259.06; 177.06; 151.00; 125.02; 83.01	Eriodictyol	FL	^38^
20	6.88	585.0881	C_27_H_22_O_15_	0.2	301.04; 283.05; 169.01; 151.00; 125.02	Quercetin 3-(2′-galloyl-α-L-arabinopyranoside)	FL	^39^
21	6.96	197.0450	C_9_H_10_O_5_	0.0	169.02; 124.02; 106.01; 78.01	Ethyl gallate	GD	^40^
22	7.03	349.0560	C_16_H_14_O_9_	0.0	197.05; 169.01; 125.02; 124.02	2,3,5,7-Tetrahydroxychroman-3-O-gallate	GD	–
23	7.72	703.1668	C_36_H_32_O_15_	0.7	541.11; 497.12; 389.10; 311.06 229.05	Fukugentin-7″-glucose	GD/FL	–
24	7.77	301.0348	C_15_H_10_O_7_	0.0	193.01; 178.99 151.00	Quercetin	FL	^41^
25	9.23	537.0821	C_30_H_18_O_10_	− 0.2	417.06; 375.05; 331.06; 159.05	Amentoflavone	FL	^42^
26	9.55	539.0980	C_30_H_20_O_10_	0.4	413.07; 387.09; 319.02; 293.05; 267.07; 251.04; 225.06; 161.02; 125.02	Volkensiflavone	FL	^43^
27	9.63	541.1133	C_30_H_22_O_10_	− 0.4	415.08; 389.10; 351.09; 311.06; 243.07; 201.06; 159.05; 125.02	Isochamaejasmin	FL	^44^

*CA* catechin, *FL* flavonoid, *GD* gallic acid derivative.

**Figure 4:**
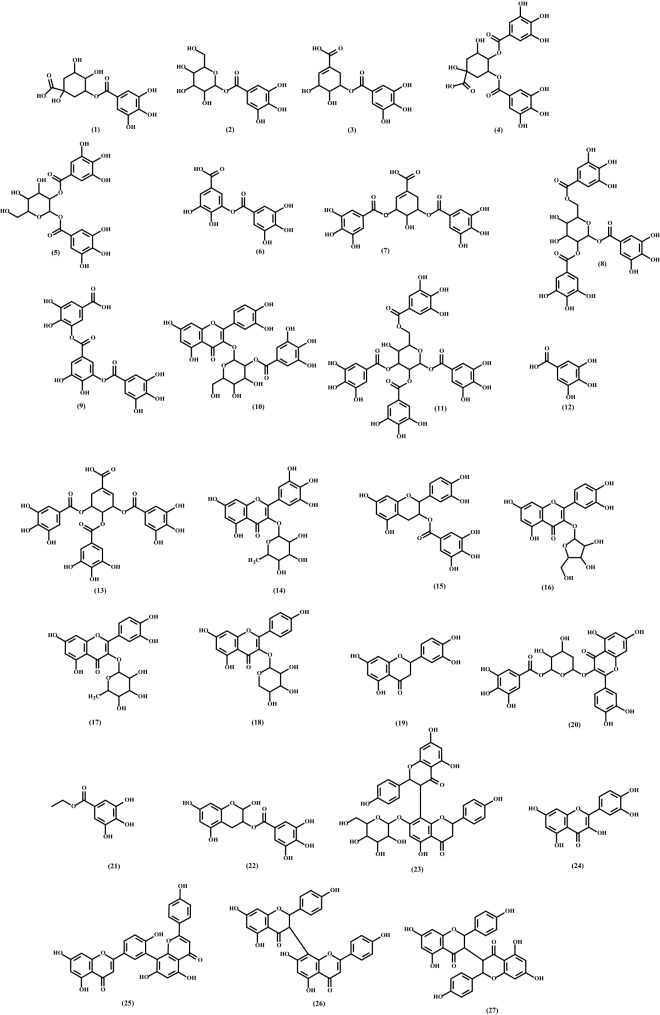
Chemical structures of the proposed compounds 1–27 present in the ethanolic extract of *Schinus terebinthifolius* (Brazilian pepper).

### Drug release from GIC-BPE composite

**Figure 5:**
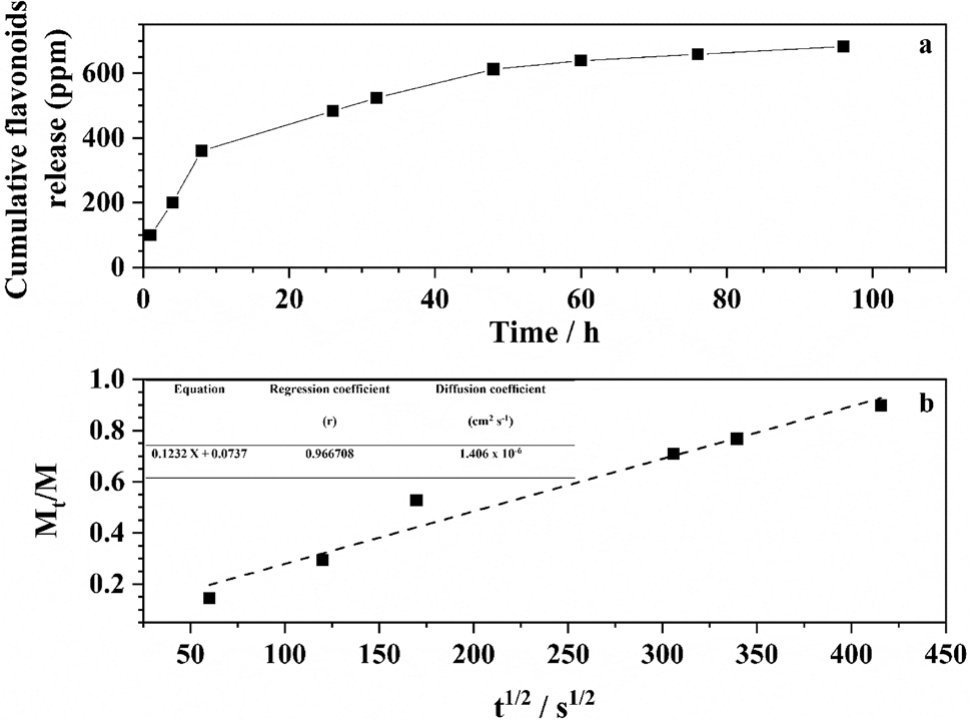
(**a**) Proportion of released flavonoid for sample GIC-BPE, (**b**) diffusion equation, regression coefficients, diffusion coefficient for the flavonoids release from GIC-BPE, and diffusion plot for flavonoids extracted from the GIC-BPE sample.

Figure 5a shows the curve of flavonoid release in two stages. The first stage corresponds to a rapid first release of the drug; in the second stage, a more controlled and slower behavior is observed until approximately 96 h.

The drug releasing profile was used to determine M_t_/M_∞_ values. The 96 h-release value was taken as a reasonable estimation of the M_∞_ value. This plot was used to determine the diffusion coefficient through the known Stefan approximation, meaning inserting into the equation: D = s^2^πl^2^/4, where s is the slope of the diffusion plot and l is the specimen thickness. The release data is shown in Fig. 5b.

Cytotoxicity testing showed that the GIC-BPE and GIC samples exhibit lower cytotoxicity than the BPE sample. Furthermore, all samples showed low cytotoxicity for MRC-5 cells.

The GIC-BPE at concentrations 16, 31, 62, 125 and 250 µg/mL showed no cytotoxic effect for MRC-5 cells, with cell viability above 70%^45^.

The extracted essential oil from the *S. terebinthifolius* is reportedly used to treat respiratory illnesses, mycosis and candida infections^46^; its activity is assigned to the high concentrations of monoterpenes, phenols and flavonoids^11^. Despite of this, to the best of our knowledge, there is no study about the use of the ethanolic extract from *S. terebinthifolius* fruit against pathogenic microorganisms of the oral cavity. In this work, we evaluated the activity of ethanolic extract from fruits *S. terebinthifolius* extract in a releasing device to form a therapeutic system to act against common pathogens in the human oral cavity. The advantage of this device is that it provides slower and longer release of the drug.

**Figure 6:**
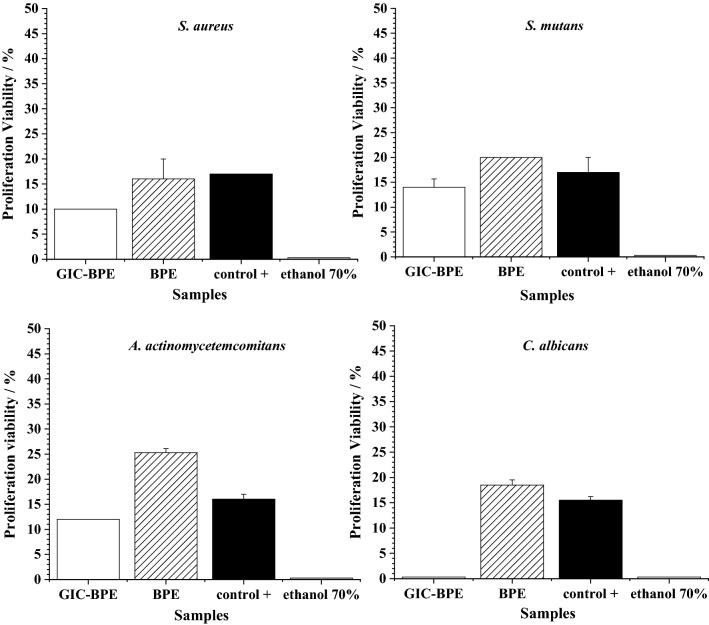
Susceptibility of samples of: a glass-ionomer—Brazilian pepper extract cylindrical pellet (GIC-PE), and blank disks soaked in ethanolic Brazilian pepper extract (BPE), positive control (chlorhexidine or nystatin for bacteria or yeast, respectively), and solvent control (ethanol 70%), against *S. aureus*, *S. mutans*, *A. actinomycetemcomitans*, and *C. albicans*. Media and standard deviation (MD ± SD), mean of three experiments.

The agar diffusion test (Fig. 6) showed that the BPE had significant antimicrobial activity against all bacterial species evaluated. The GIC-BPE sample had lower antimicrobial activity than the BPE sample and has no antimicrobial activity against *Candida albicans*. No inhibition zone was observed when ethanol was tested alone.

## Discussion

The ATR-FTIR spectrum for the GIC-BPE sample (Fig. 2c) shows bands of GIC and of BPE, which indicates that the Brazilian pepper extract was incorporated into the ionomer. In addition, there was overlapping of some bands. Despite this, multiple bands of the BPE sample and some bands of the GIC sample disappeared in the GIC-BPE sample indicating that a probable intermolecular interaction exists between the glass-ionomer and functional groups of flavonoid compounds from BPE.

The first weight loss for the GIC sample was attributed to the removal of water from the surface. This stage was reflected in the DTG curve in this region (Fig. 3a). The mass loss at the higher temperature represents not only the degradation of any evolving polyalkenoate polymer but also the decomposition of unreacted components^47–49^.

For the BPE sample (Fig. 3b), the mass loss event in the first zone (30–118.40 °C) is assignable to the loss of water and light volatile compounds in the sample (13.78%) and a peak on the DTG curve was observed around 94.1 °C. The other zone (118.40–272.17 °C) is associated with the thermal decomposition of carbohydrates and other organic compounds present in the plant^50^. In the third zone, the organic materials were gradually released (19.35%), resulting in a large mass loss and the formation of the main pyrolytic products. As shown in the derivative weight loss (DTG), several stages of decomposition could be distinguished. Studies of phytochemicals identified the presence of tannins, flavonoids, and terpenoids in the species under study. In the last stage, between 370.42 and 600 °C, these carbonaceous matters decomposed^51^. More than 43% of the weight loss of the sample occurred in this stage.

While the GIC sample lost about 26% by mass up to 600 °C, the GIC-BPE sample lost about 84%. Most of this difference in mass loss is due to the pepper extract incorporated in the GIC. Interestingly, although the GIC-BPE sample has a large amount of BPE, the peak shown on the DTG curve of the GIC-BPE sample is at a temperature similar to that of the GIC sample.

More insights about the thermal decomposition peaks can be obtained from DTA curves (Fig. 3). The endothermic peak of the DTA profile for the BPE sample (Fig. 3b) is due to a dehydration event corresponding to the first mass loss of the TG curves. The exothermic event is likely due to the degradation and oxidation of organic matter and corresponds with the fourth mass loss observed in the TG curve. These results have been observed by other authors with starches from several botanical origins and treatments^52,53^. The DTA curve for the GIC-BPE sample (Fig. 3c) shows the presence of an exothermic peak at 470 °C. It may be that the exothermic peak of the BPE sample (514 °C) has shifted to a lower temperature (470 °C). This decrease in the thermal stability of BPE suggests a chemical interaction between the components of the extract (BPE) with the glass-ionomer, confirming the ATR-FTIR result.

The compressive strength of the GIC-BPE sample was lower than that of the GIC sample. Similar results were reportedly found for other additives, including propolis, antimicrobials and bioactive glasses^16,54,55^. Whatever its chemical origin is, this trend to lower its therapeutic strength is widespread and is characteristically associated to the inhibition of the setting reaction due to the presence of the additive. It seems that the reducing mechanical properties of the cements, on adding additives, is due to their concentrations and to even small amounts of the additive. This decrease appears to be related to chemical interactions between the drug and the glass ionomer chemical components. Thus, the cationic compounds based on quaternary ammonium salts, such as benzalkonium chloride and cetyl pyridinium chloride, have a particular ability to do this via interaction with the poly(acrylic acid) component^56,57^. However, this effect has also been observed for neutral species, such as methanol^58^, 2-hydroxethyl methacrylate^58^ and sodium chloride^59^. The chemical compounds of the ethanolic extract of *S. terebinthifolius* are likely to interact with the glass ionomer. This was evidenced from the FTIR and thermogravimetric results.

Several factors can determine the chemical composition of a plant, such as availability of nutrients, and organic matter in the soil^60^. Because of this, a chemical analysis was performed by UPLC–QTOF–MS/MS (Fig. 4 and Table 2) to identify the components of the ethanolic extract so obtained. The characterized compounds were classified into three groups: gallic acid derivative, flavonoid and catechin. These data are in agreement with the previous phytochemical analysis, since galloyl derivatives can also be classified as tannins.

The galloyl derivatives fragmentation pattern is mainly characterized by ions at m/z 191.06, 169.01 and 125.02 (Fig. S60). The loss of gallic acid and quinic acid structures is related to the first and second fragment ions, respectively. Finally, the loss of CO_2_ corresponds to the last ion^61^. On the other hand, flavonoids do not show a fragmentation pattern since it can vary according to their classification. In the Fig. S61 is represented an example of fragmentation from flavonoid that was not found in the literature but that was proposed as fukugentin-7″-glucose. In addition, a proposed fragmentation of catechin 3-O-gallate is described in the Fig. S62.

Among the suggested compounds, gallic acid, quercetin and quercitrin have already been reported for *Schinus lentiscifolius* that showed a broad spectrum of antimicrobial activity^62^. In this same way, ethanolic extract from fruits and leaves of *S. terebinthifolius* showed high potential against *E. coli* and this data can be related to the presence of ethyl gallate, which was one of the main compounds reported^63^. Other proposed substances, such as myricitrin^64^, catechin 3-O-gallate^65^, avicularin^66^, and amentoflavone^67^ also demonstrated action against microorganisms. Thus, all of these data support the findings of our work.

The curve shows a rapid first release of flavonoids (Fig. 5). It is suggested that this rapid initial release occurs as some drugs became trapped on the surface of the support, during its preparation^68^, to be freed immediately upon activation in a proper medium. In a second stage, a more controlled and slower behavior is observed. This slower release suggests an interaction between the GIC and the flavonoids, as it is evidenced by the FTIR, thermogravimetric and mechanical data.

The resulting diffusion plot relating M_t_/M_∞_ and $\sqrt t$ was found to be linear for about the first 48 h, indicating that the release occurred through a diffusion-governed mechanism following the Fick’s law of diffusion (Fig. 5b). Several studies have reported the antimicrobial compounds being incorporated into glass-ionomer cements. Diffusion coefficients values from some of these studies were: 6.16 × 10^–9^ and 1.39 × 10^–9^, for 3% the cetylpyridinium chloride and 3% benzalkonium^69^; 4.4 × 10^–8^ for the 1% sodium fusidate^70^ and 3 × 10^–7^ for the 10% chlorhexidine cm^2^ s^−1^^71^ release from glass ionomer. Several reportedly results^70,71^ have shown that increasing the concentration of a given drug tends to decrease the diffusion coefficient. As a comparatively remarkable result, in this work, the concentration of flavonoids in the GIC-BPE sample is approximately 0.5% and the diffusion coefficient was found to be 1.406 × 10^−6^ cm^2^ s^−1^.

The results of cytotoxicity these tests showed that the viability of the MRC-5 cells was BPE concentration dependent, and the cytotoxic concentration for 50% of the cells (CC_50_) was 2696, 701.3 and 154.8 µg/mL for GIC, GIC-BPE and BPE, respectively.

The lower antimicrobial activity of GIC-BPE sample, compared to BPE sample, can be explained by the fact that in the GIC-BPE, the BPE is incorporated into the ionomer, which makes the delivery slower.

According to Jakobek^72^, the detected flavonoids and phenolic compounds in plant extracts act as antimicrobial agents against various human pathogens. The mechanism of action of polyphenols on microorganisms is still poorly understood, and some authors suggest that polyphenol acts by reducing the iron availability, inhibiting protein expression, changing the cell membrane permeability and fluidity^73–75^.

The GIC-BPE sample showed antimicrobial activity at non-cytotoxic concentrations to human fibroblasts cells. Therefore, the obtained results suggest that this extract can be an efficient alternative for the treatment of infections of the oral cavity, such as stomatitis, dental cavities and periodontitis caused by *S. aureus*, *S. mutans*, *A. actinomycetemcomitans*, and *C. albicans*.

## Material and methods

### Material

All GIC samples were prepared from Maxxion C. All other reagents (analytical grade) were used as received.

### Preparation of ethanolic extracts from the *Schinus terebinthifolius* Raddi fruits (BPE) and from the GIC-BPE sample

Fruits of *S. terebinthifolius* Raddi were collected in Ouro Preto, Minas Gerais State, Brazil (20° 23′ 8″ S and 43° 30′ 13″ W) in April 2019. A voucher specimen was identified and deposited at the UFOP José Badini herbarium under the code OUPR31536.

Extraction involved macerating the fruits (1.08 kg) at room temperature with ethanol 95% (2 L, 2 consecutive extractions over 24 h). The extracted solutions were filtered and submitted to vacuum evaporation to afford 123.64 g of ethanolic crude extract.

The GIC was prepared according to the manufacturer's instructions by mixing the powder and liquid in a 1:1 ratio. The drug-free samples were labelled GIC.

The BPE was incorporated into the glass-ionomer powder and mixed for 15 s with a spatula. Then, the liquid phase of the glass-ionomer was added to the mixture and homogenized to slurry. The resulting paste was then dried in an oven at 37 °C for 1 h. The amount of BPE initially added was approximately 38 wt%. This sample was labelled GIC-BPE.

### Characterization techniques of the samples

The morphology of the GIC and GIC-BPE samples was studied by SEM (Jeol JSM 6010LA). Prior to analysis, the samples were fastened to a sample holder with the help of a double carbon ribbon and covered with carbon. The sample structure information was collected by attenuated total reflectance Fourier transform infrared spectroscopy (ABB Bomen MB 3000 ATR-FTIR spectrometer, Quebec, Canada) at a resolution of 4 cm^‒1^ from 500 to 4000 cm^‒1^ and 32 scans per sample. The thermogravimetric, differential thermogravimetric and differential thermal analysis studies were carried out in a Shimadzu TG-60 thermal analyser from 25 to 700 °C at 10 °C/min in air. Al_2_O_3_ was used as standard. The compressive strength test of the GIC and GIC-BPE samples was performed according to ISO standardization^76^ (EZ Test, Shimadzu). The compressive strength of the samples was calculated using the equation P/πr^2^, where: P = load at fracture, r = the radius of the sample cylinder and π (constant) = 3.14; the values obtained were expressed in MPa. Correlations between values were analysed using a student t test, with a significance level of 5%.

Chemical tests were carried out on the BPE to identify the phytoconstituents, i.e., alkaloids, anthraquinones, flavonoids, carbohydrates, saponins, tannins and terpenoids, as per the standard procedure^77^. In the chromatographic analyses the BPE was subject to clean-up process and two fractions at 50 and 100% acetonitrile (ACN) (F50ACN and F100ACN) were obtained at 200 ppm concentration (v/v) using reverse phase (1.0 g C-18 cartridge) and mobile phase ACN:H_2_O (1:1). The chromatographic separation was carried out on an Agilent Zorbax SB-C18 column (3.0 × 50 acid and water—0.1% formic acid such mobile phase at flow rate of 0.3 mL min^−1^ after injection of 3 µL of analytical solutions). The chromatographic analyzes were performed at gradient mode (5–100% of ACN during 17 min and kept under 100% ACN during 2 min). The MS1 and MS2 data were acquired in positive and negative mode and in Auto-MS/MS mode for the fragment ions by Agilent 6545 Q-TOF (Agilent Technologies Inc., Santa Clara, CA) equipped with electrospray ionization (ESI) source. The operational source parameters for TOF–MS mode were: capillary voltage of 3000 V, skimmer voltage of 65 V, fragmentation voltage of 110 V, nebulizer gas pressure of 35 psi, dry gas flow of 12 L min^−1^, gas temperature of 320 °C, sheath gas flow of 10 L min^−1^, sheath gas temperature of 300 °C, acquisition rate of 3 spectrum per second, and resolution of 32,000. The auto MS/MS mode was performed with table of collision energy voltage of 12 spectra per cycle, using 150–500 Da (10–15 eV), 500–1000 Da (15–30 eV), and 1000–1500 Da (30–50 eV) at medium energy of collision, whereas to high energy of collision was 150–500 Da (25–40 eV), 500–1000 Da (40–55 eV), and 1000–1500 Da (55–70 eV). MassHunter workstation software was used for acquisition and processing of the data and the ionized molecule [M−H]^−^ obtained in the TOF–MS mode were calculated.

The measurement of total phenolic content was made by the Folin-Ciocauteu method according to Bonoli et al. 2004^78^ and, after incubation for 2 h, the absorbance was read at 650 nm in a microplate reader (Molecular Devices). The total phenolic content was quantified using a standard calibration curve of gallic acid (2.8–180.0 µg/mL; r^2^ = 0.9622; y = 0.0075x + 0.1854). The experiment was performed in triplicate and the results were expressed as mg of gallic acid equivalents per 1 g of sample (mg GAE/g).

The measurement of total flavonoids was made by the aluminium chloride (AlCl_3_) colorimetric method according to Chang et al.^79^. The absorbance was read after incubation for 40 min in a microplate reader (Molecular Devices) at 405 nm. The total flavonoids were quantified using a standard calibration curve of quercetin (1.0–32.0 µg/mL; r^2^ = 0.9749; y = 0.0012x + 0.0371). The experiment was performed in triplicate and the results were expressed as mg of quercetin equivalents per 1 g of sample (mg QE/g).

### Drug release

To perform the flavonoid delivery test, it was necessary to prepare GIC-BPE cylindrical pellets. The pellets were prepared by compressing approximately 0.25 g of the powder, at a 0.5 ton loading for 1 min^14,80,81^.

As already established for the release profile test^14,80,81^, the pellets were placed in glass vials, immersed in 3 mL of a body fluid simulating solution (SBF)^82^ and incubated at 37 °C. After the intervals of 1, 4, 8, 26, 32, 48, 60, 76 and 96 h, all the solution was removed for analysis, and the pellets were immersed in 1 mL of ethanol for 10 s. Following ethanol removal, a new SBF solution was placed in the vials containing the pellets.

The amount of flavonoids extracted from BPE sample was measured using a method derived from that of Dowd^80,83^. Preparation of the solutions for spectrophotometer analysis consisted of transferring the following to a Falcon tube: 1 mL of the SBF solution removed from the vial containing the pellet, 0.5 mL of the ethanol used to wash the pellet and 1 mL of AlCl_3_ 2% in ethanol. After 10 min the Falcon tubes containing the test solutions were centrifuged at 2500 rpm for 5 min. The test solution turned yellow, and the absorbance was read at 420 nm.

The flavonoid concentration was determined by ultraviolet–visible absorption spectroscopy (UV–Vis) at 420 nm (Thermo Scientific-Genesys 840 spectrophotometer). The calibration curve of the flavonoid concentration was established by using ethanol solutions with known concentrations of quercetin.

### Determination of cytotoxicity—sulforhodamine B (SRB) colorimetric assay

Human fibroblast MRC-5 cells, cultivated in RPMI 1640 medium (Sigma), were distributed in a 96-well microtiter plate using a density of 5 × 10^4^ cell/well and incubated at 37 °C with 5% CO_2_ for 24 h. Cells were treated with the sample dissolved in RPMI 2% DMSO for 24 h, at concentrations ranging from 16 to 1000.00 µg/mL. Cell viability was evaluated by sulforhodamine B assay (SRB)^84^. After 24 h incubation the media was removed and cells were fixed with cold 20% trichloroacetic acid for 1 h at 4 °C. The microtiter plate was washed with distilled water and dried. Thereafter, fixed cells were stained for 30 min with 0.1% SRB dissolved in 1% acetic acid. The plate was washed again with 1% acetic acid and allowed to dry before 200 µL of 10 mM TRIS buffer (pH 10.5) was added to remove unbound dye. This was done at room temperature for ~ 30 min. Sample absorbance was read in the spectrophotometer (490 nm) and the 50% cytotoxic concentration (CC_50_) was defined as the concentration that reduced cell viability by 50% when compared to untreated controls. CC_50_ values were calculated by a non-linear regression equation using GraphPad Prism software.

The definition of the cytotoxicity used was: CC_50_ < 1.0 μg/mL—high cytotoxicity; CC_50_ 1.0–10.0 μg/mL—moderate cytotoxicity; CC_50_ 10.0–20.0 μg/mL—mild cytotoxicity; and CC_50_ > 20 μg/mL—non‐cytotoxic^85^.

### Antimicrobial activity

The antimicrobial activity of BPE, 70% ethanol, and the composite (GIC-BPE) were studied against two Gram-positive bacteria, *S. aureus* (ATCC 12692) and *S. mutans* (ATCC 25175), microorganisms causing caries; one Gram-negative bacteria, *A. actinomycetemcomitans* (ATCC 33384), microorganism periodontopathogens; as well as *C. albicans* yeast (ATCC 18804), which causes oral lesions. All microorganisms were donated by the Collection of Reference Microorganisms on Health Surveillance from Oswaldo Cruz Foundation, RJ, Brazil. The susceptibility tests were performed using agar diffusion^86^. A 0.1 mL aliquot of 24 h cultures of *C. albicans* was incubated at 37 °C in Sabouraud dextrose broth (Difco), corresponding to 5.0 turbidity units on the McFarland standard (1.5 × 10^8^ UFC/mL), and seeded in 30 mL Sabouraud dextrose agar (Difco). A 0.1 mL aliquot of overnight cultures of *S. mutans* strains incubated at 37 °C in brain heart infusion (BHI) (Difco), corresponding to 0.5 turbidity units on the McFarland standard, was plated onto 30 mL Mitis salivarius bacitracin agar (Difco). *S. aureus* and *A. actinomycetemcomitans* species were incubated overnight at 37 °C and a 0.1 mL aliquot of each was plated onto 30.0 mL blood agar (Difco) supplemented with hemin/menadione (Sigma). Microorganisms were incubated at 37 °C for 18 h in anaerobic system (Difco).

The antimicrobial activity was measured by the inhibition zones produced. The inoculum was initially standardized to 10^8^ CFU/mL (50% transmittance at 580 nm), by transferring colonies from the nutrient agar to sterile saline, and 200 μL of the suspension was homogeneously placed on the surface of 50 mL Mueller–Hinton agar in a 150 mm Petri dish. GIC-BPE samples, measuring 6.0 mm in diameter, were planted on the surface of the agar and incubated at a temperature of 37 °C, in an environment with an atmosphere of 5% CO_2_. Sterile blank disks (CECON) were soaked in 20 µL of the isolated ethanolic BPE (46 µg/mL) and applied to the agar surface previously seeded with the microorganisms. Additionally, each plate carried control disks: solvent control (20 μL sterile ethanol), and discs containing 10 µg/mL of chlorhexidine (Sigma) for bacteria and 20 µg/mL of nystatin (Sigma) for yeast that were used as inhibition growth positive controls for comparison with the GIC-BPE sample. After 24 h of incubation at 37 °C, the diameters of the inhibition zones were measured and compared. Tests were performed in triplicate.

## Conclusion

In this work we studied the physical–chemical, cytotoxic, antibacterial and mechanical properties of GIC-BPE and we compared the results obtained with those of GIC, frequently used in dental restorative clinics. For the GIC-BPE sample, we also evaluated the flavonoid release profile. The ATR-FTIR results can indicate that the flavonoids present in BPE were incorporated into the ionomer, and the study of the flavonoid release profile showed that the flavonoids were practically all released within 60 h. FTIR and thermal analysis results indicated that an intermolecular interaction exists between the glass-ionomer and BPE. This interaction can explain the slight decrease in mechanical properties of the GIC-BPE sample compared to GIC (176.60 ± 6.46 MPa for GIC vs. 161.17 ± 5.01 MPa for GIC-BPE) and the smaller thermal stability of the GIC-BPE samples when compared with the free extract samples (BPE). The results of cytotoxicity and antimicrobial tests showed that the GIC-BPE has antimicrobial activity at non-cytotoxic concentrations to human fibroblasts cells. The results suggest that the prepared composite may represent a new therapeutic option as an alternative agent available for endodontic treatment. This product has a low cost and is an interesting alternative for use in dental treatment, especially in developing countries where the majority of people use the public health support system.

## Supplementary Information

Supplementary Information
