# Supplementary material for: Preparation of glass-ionomer cement containing ethanolic Brazilian pepper extract (Schinus terebinthifolius Raddi) fruits: chemical and biological assays
Source: Sci Rep. 2020 Dec 18;10:22312. doi: 10.1038/s41598-020-79257-3 (PMC7749175; doi:10.1038/s41598-020-79257-3)
Supplement: Supplementary file 1 — Supplementary Information [file 41598_2020_79257_MOESM1_ESM.docx]

**SUPPLEMENTARY MATERIAL**

**Preparation of glass-ionomer cement containing ethanolic Brazilian pepper extract (*Schinus terebinthifolius* Raddi) fruits: chemical and biological assays**

**Isabelle C. Pinto^1^, Janaína B. Seibert^2^, Luciano S. Pinto^2^, Vagner R. Santos^3^, Rafaela F. de Sousa^1^, Lucas R.D. Sousa^1,4^, Tatiane R. Amparo^5^, Viviane M.R. dos Santos^1^, Andrea M. do Nascimento^1^, Gustavo Henrique Bianco de Souza^5^, Walisson A. Vasconcellos^3^, Paula M.A. Vieira^4^, Ângela L. Andrade^1,^***

**
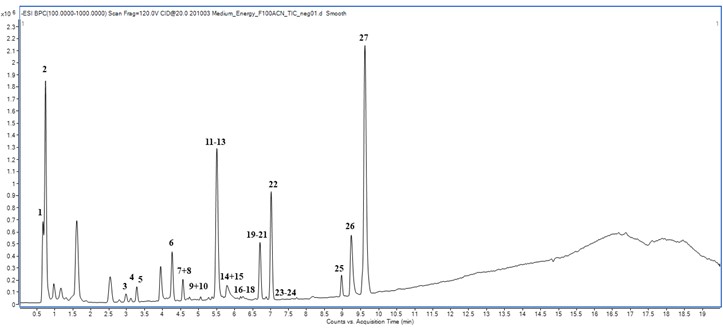
**

**Figure S1.** Chromatogram of the ethanolic Brazilian pepper extract (BPE).


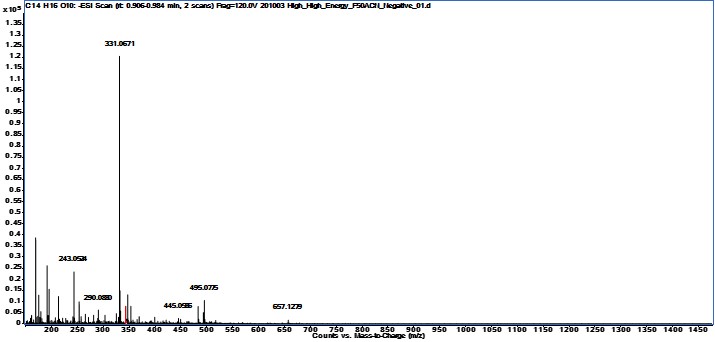


**Figure S2.** MS^1^ spectra data of compound **1** [M-H]^-^=343.0667.


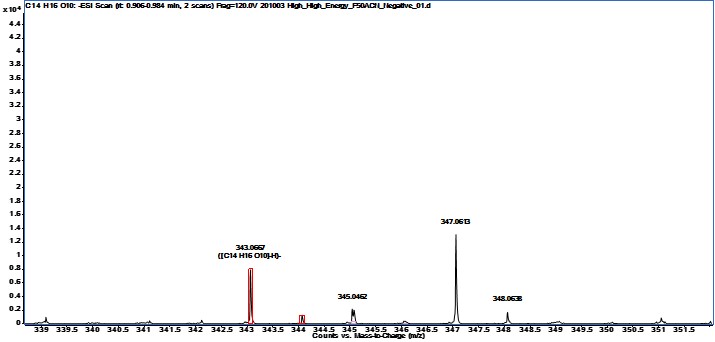


**Figure S3.** Expansion of MS^1^ spectra data of compound **1** [M-H]^-^=343.0667.


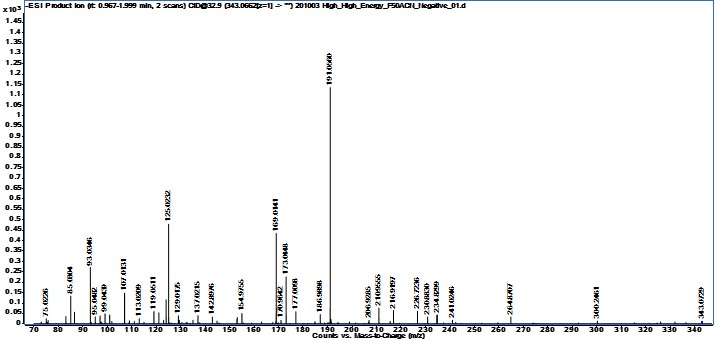


**Figure S4**. MS^2^ spectra data of compound **1** [M-H]^-^=343.0667.


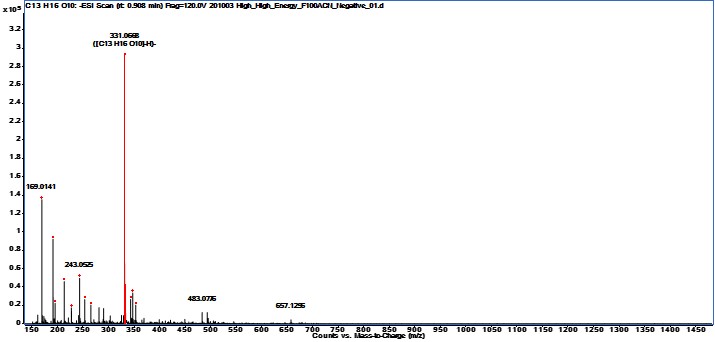


**Figure S5.** MS^1^ spectra data of compound **2** [M-H]^-^=331.0668.


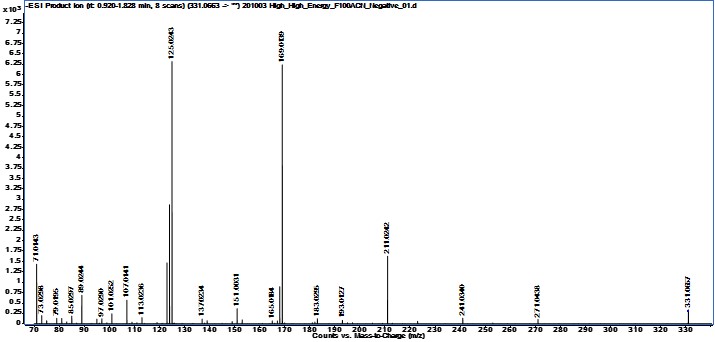


**Figure S6.** MS^2^ spectra data of compound **2** [M-H]^-^=331.0668.

**
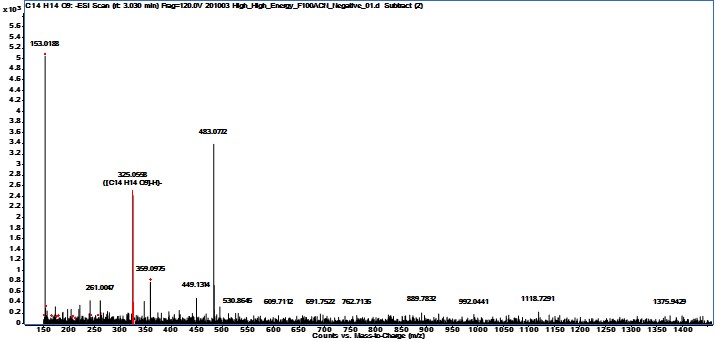
**

**Figure S7.** MS^1^ spectra data of compound **3** [M-H]^-^=325.0558.


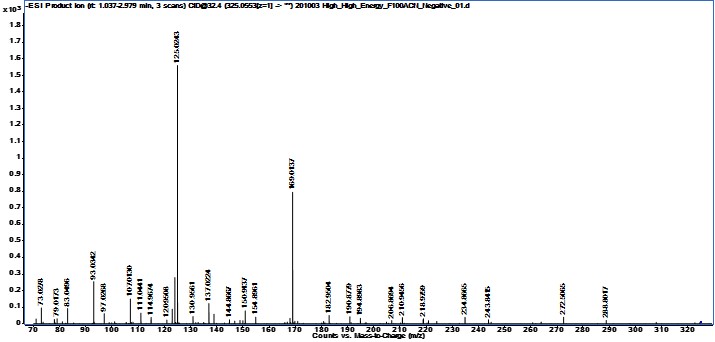


**Figure S8.** MS^2^ spectra data of compound **3** [M-H]^-^=325.0558.


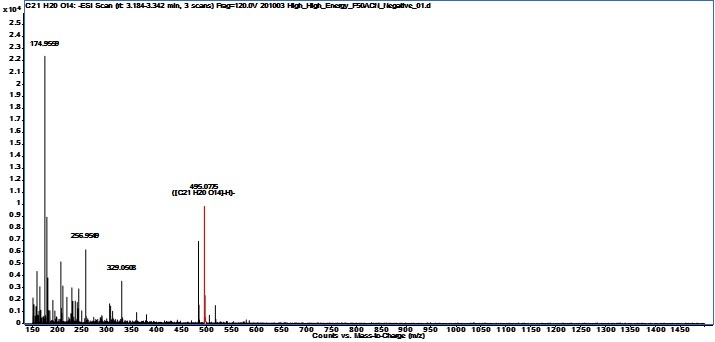


**Figure S9.** MS^1^ spectra data of compound **4** [M-H]^-^=495.0775.


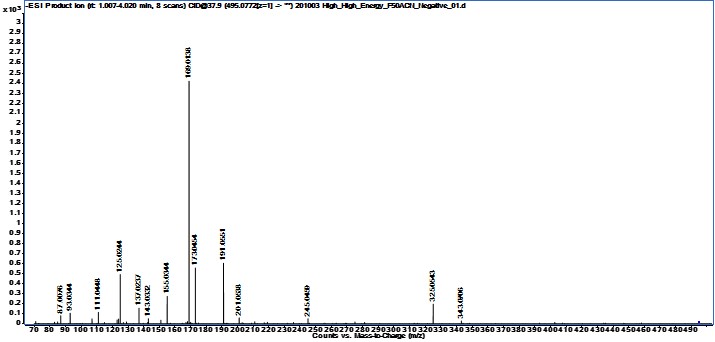


**Figure S10.** MS^2^ spectra data of compound **4** [M-H]^-^=495.0775.

**
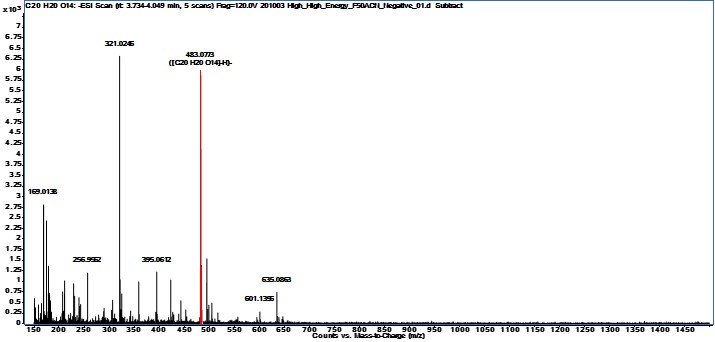
**

**Figure S11.** MS^1^ spectra data of compound **5** [M-H]^-^=483.0773.


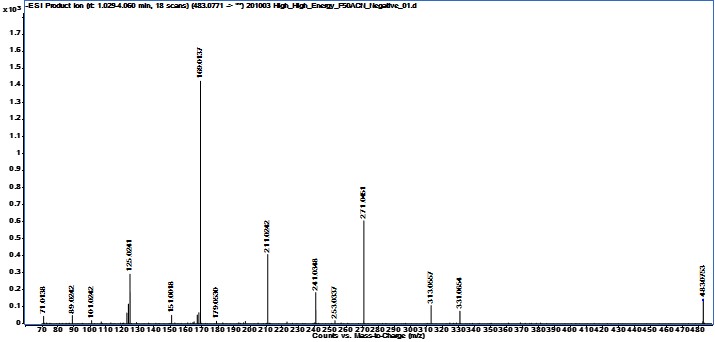


**Figure S12.** MS^2^ spectra data of compound **5** [M-H]^-^=483.0773.


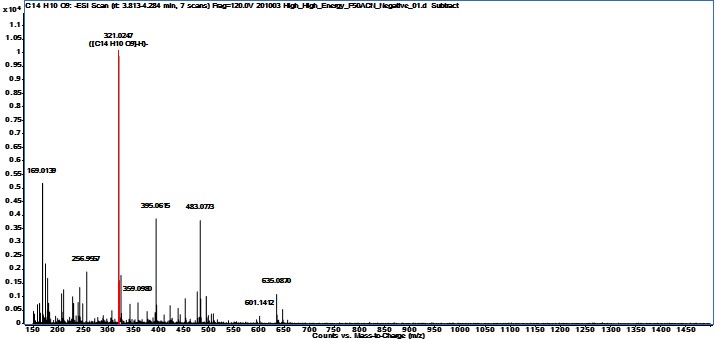


**Figure S13.** MS^1^ spectra data of compound **6** [M-H]^-^=321.0247.


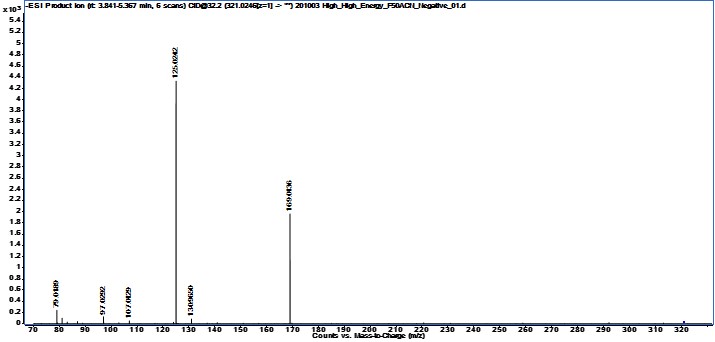


**Figure S14.** MS^2^ spectra data of compound **6** [M-H]^-^=321.0247.


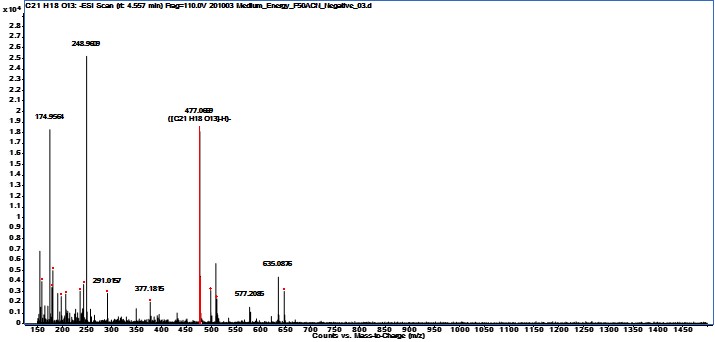


**Figure S15.** MS^1^ spectra data of compound **7** [M-H]^-^=477.0669.


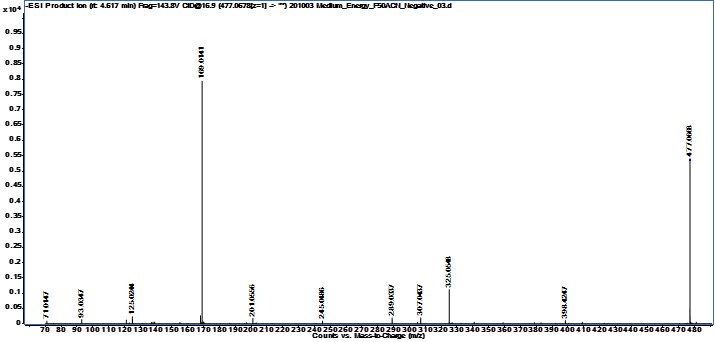


**Figure S16.** MS^2^ spectra data of compound **7** [M-H]^-^=477.0669.


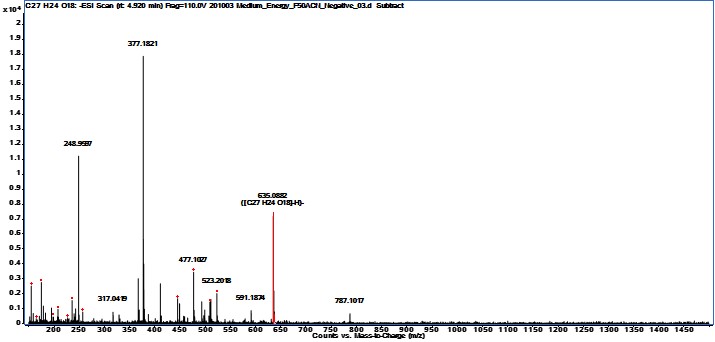


**Figure S17.** MS^1^ spectra data of compound **8** [M-H]^-^=635.0882.


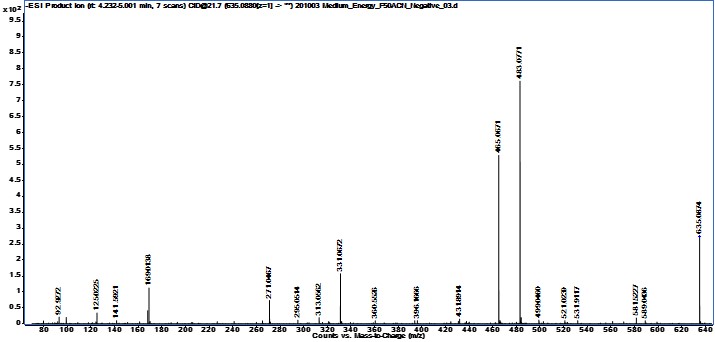


**Figure S18.** MS^2^ spectra data of compound **8** [M-H]^-^=635.0882.


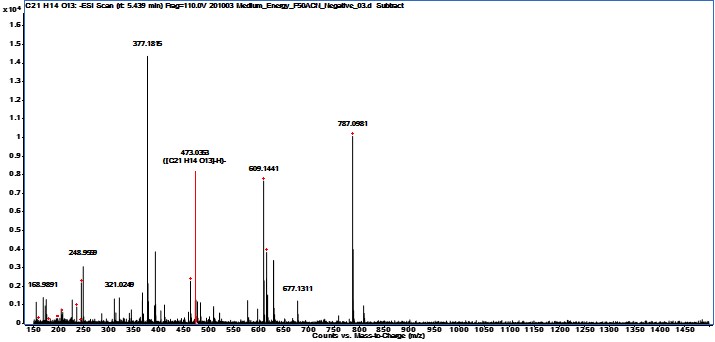


**Figure S19.** MS^1^ spectra data of compound **9** [M-H]^-^=473.0353.


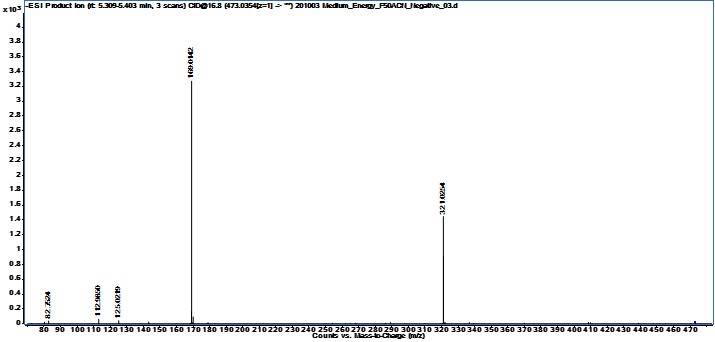


**Figure S20.** MS^2^ spectra data of compound **9** [M-H]^-^=473.0353.


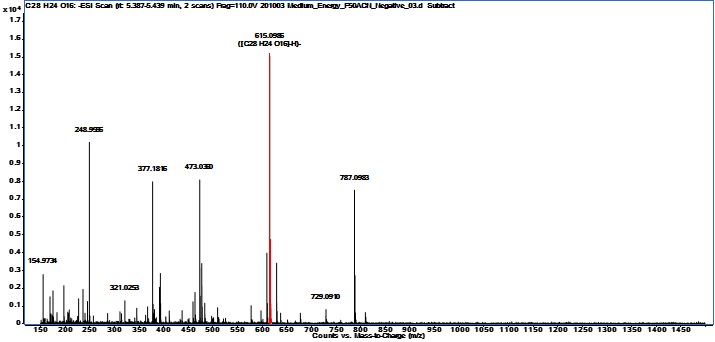


**Figure S21.** MS^1^ spectra data of compound **10** [M-H]^-^=615.0986.


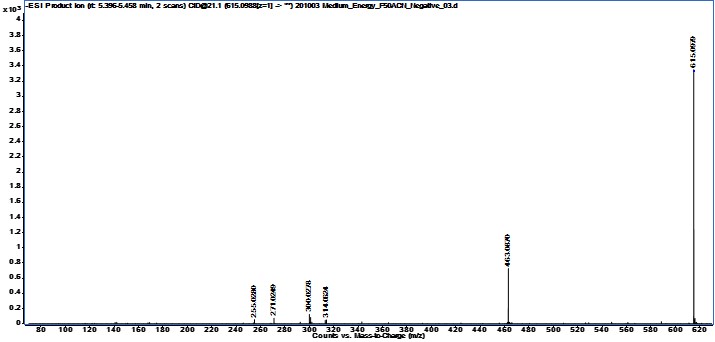


**Figure S22.** MS^2^ spectra data of compound **10** [M-H]^-^=615.0986.


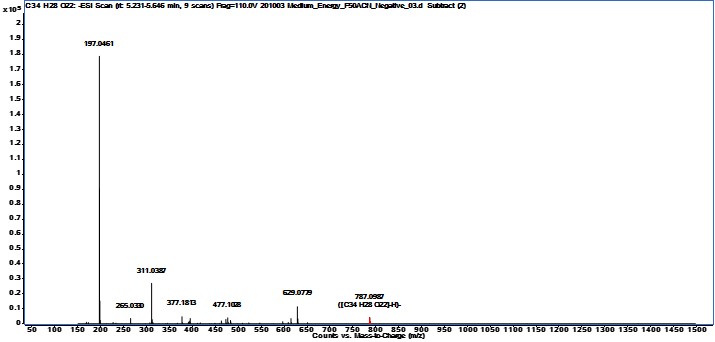


**Figure S23.** MS^1^ spectra data of compound **11** [M-H]^-^=787.0987.


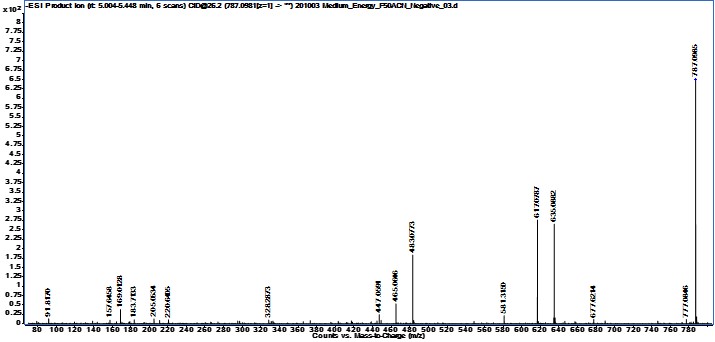


**Figure S24.** MS^2^ spectra data of compound **11** [M-H]^-^=787.0987.


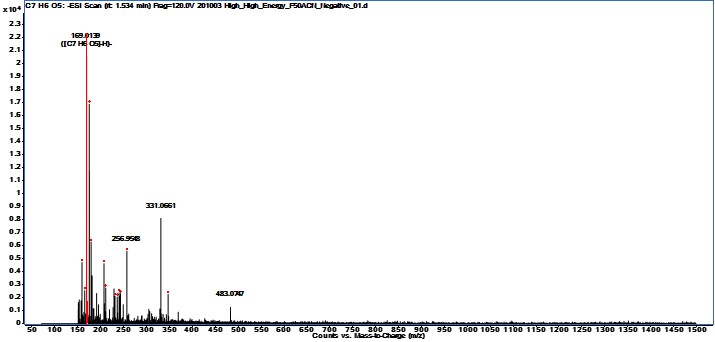


**Figure S25.** MS^1^ spectra data of compound **12** [M-H]^-^=169.0139.


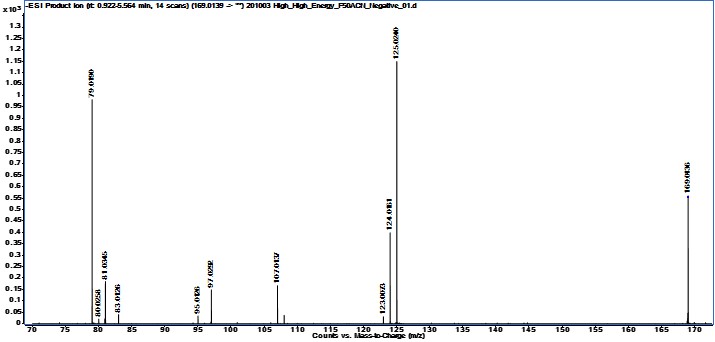


**Figure S26.** MS^2^ spectra data of compound **12** [M-H]^-^=169.0139.


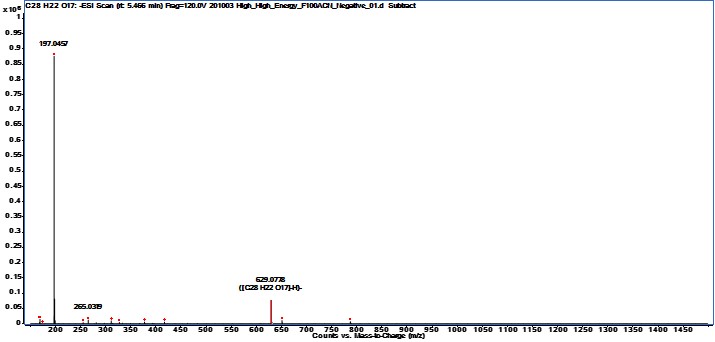


**Figure S27.** MS^1^ spectra data of compound **13** [M-H]^-^=629.0778.


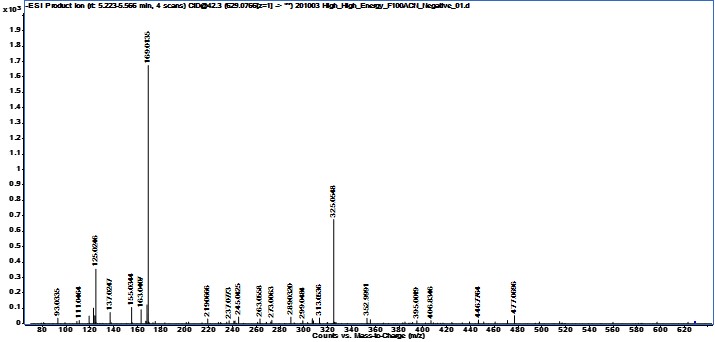


**Figure S28.** MS^2^ spectra data of compound **13** [M-H]^-^=629.0778.


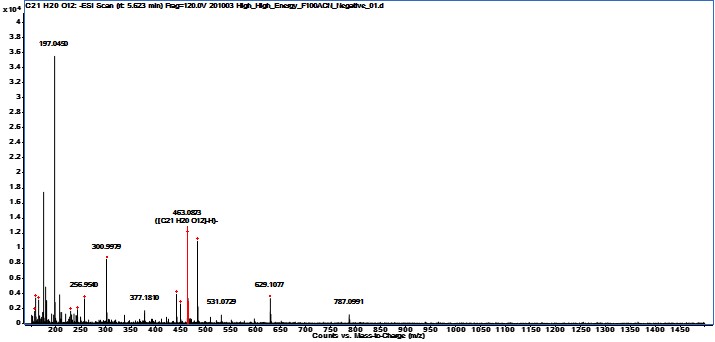


**Figure S29.** MS^1^ spectra data of compound **14** [M-H]^-^=463.0873.


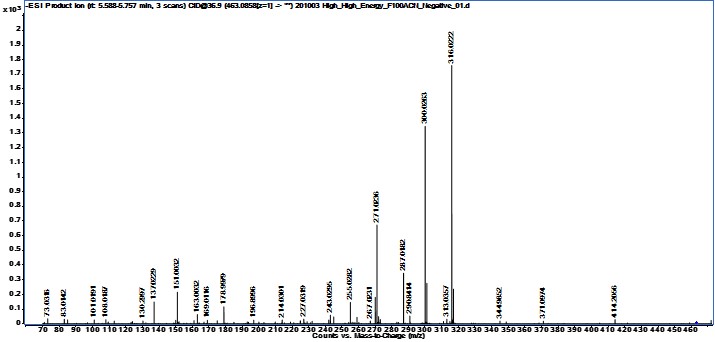


**Figure S30.** MS^2^ spectra data of compound **14** [M-H]^-^=463.0873.


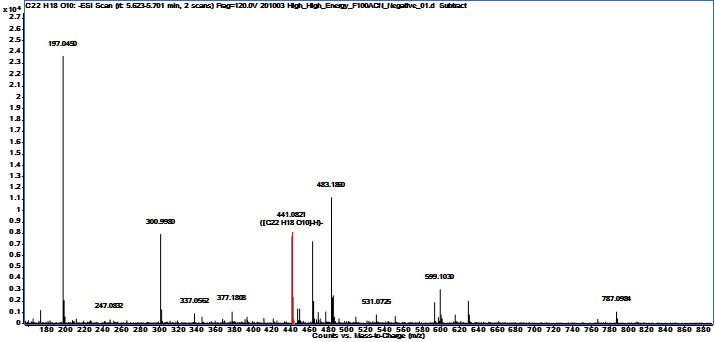


**Figure S31.** MS^1^ spectra data of compound **15** [M-H]^-^=441.0821.


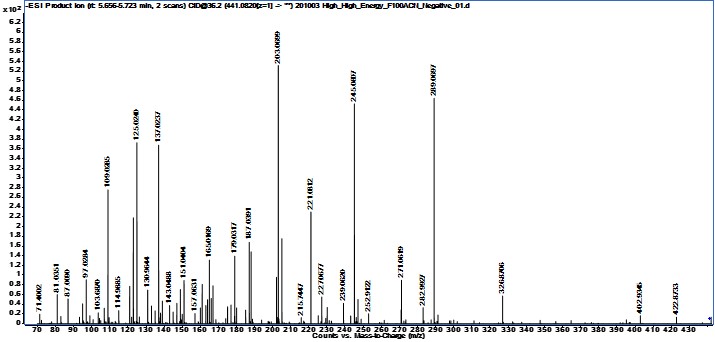


**Figure S32.** MS^2^ spectra data of compound **15** [M-H]^-^=441.0821.


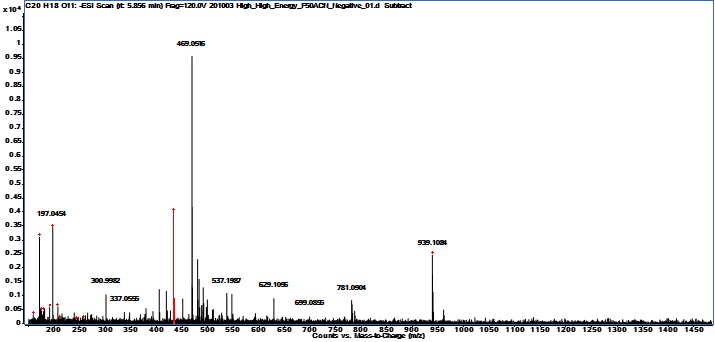


**Figure S33.** MS^1^ spectra data of compound **16** [M-H]^-^=443.0774.


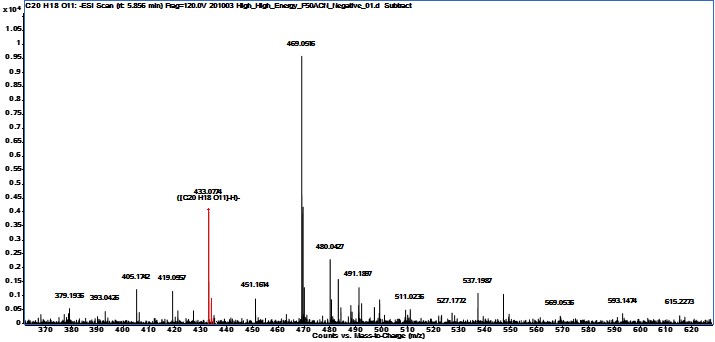


**Figure S34.** Expansion of MS^1^ spectra data of compound **16** [M-H]^-^=433.0774.


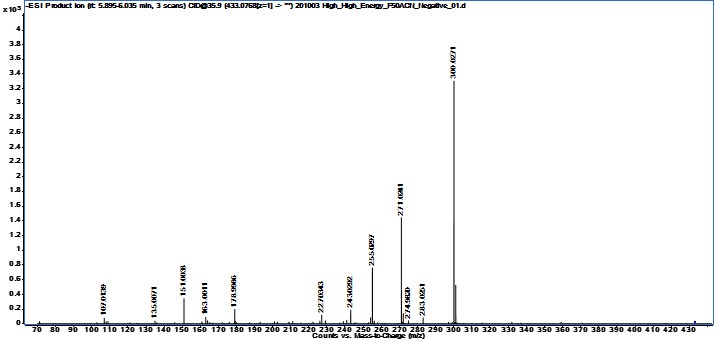


**Figure S35.** MS^2^ spectra data of compound **16** [M-H]^-^=433.0774.


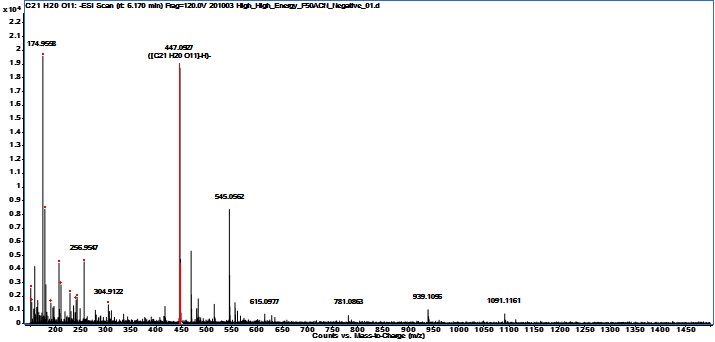


**Figure S36.** MS^1^ spectra data of compound **17** [M-H]^-^=447.0927.


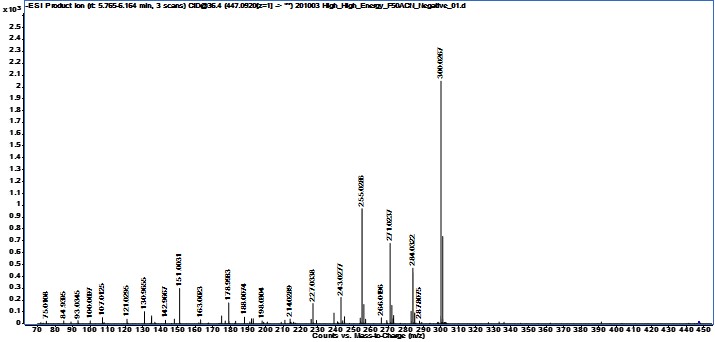


**Figure S37.** MS^2^ spectra data of compound **17** [M-H]^-^=447.0927.


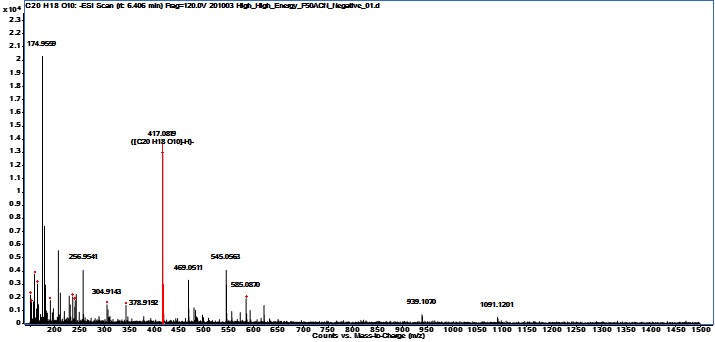


**Figure S38.** MS^1^ spectra data of compound **18** [M-H]^-^=417.0819.


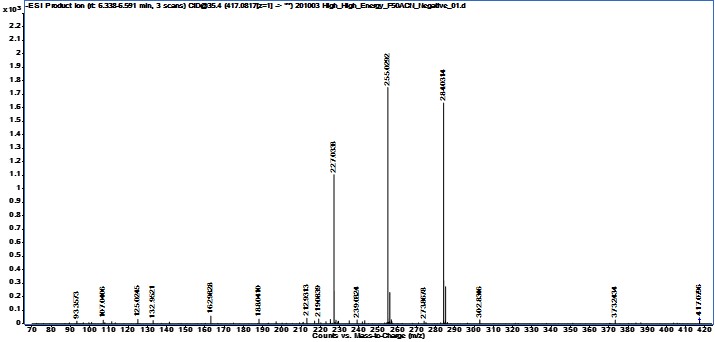


**Figure S39.** MS^2^ spectra data of compound **18** [M-H]^-^=417.0819.


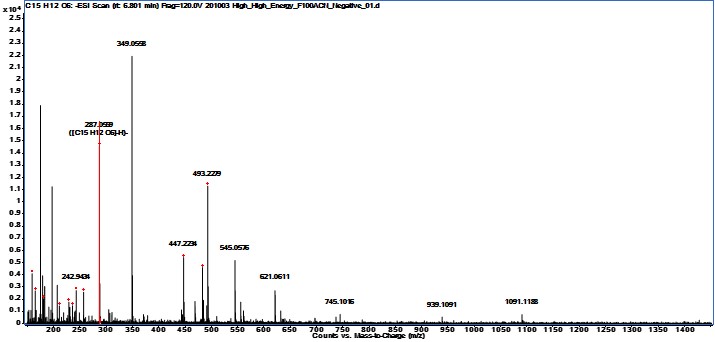


**Figure S40.** MS^1^ spectra data of compound **19** [M-H]^-^=287.0559.


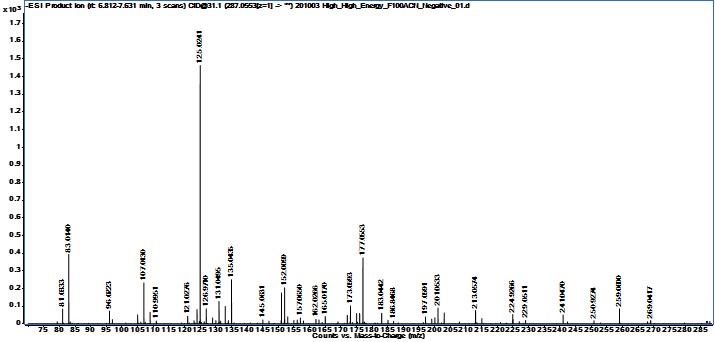


**Figure S41.** MS^2^ spectra data of compound **19** [M-H]^-^=287.0559.


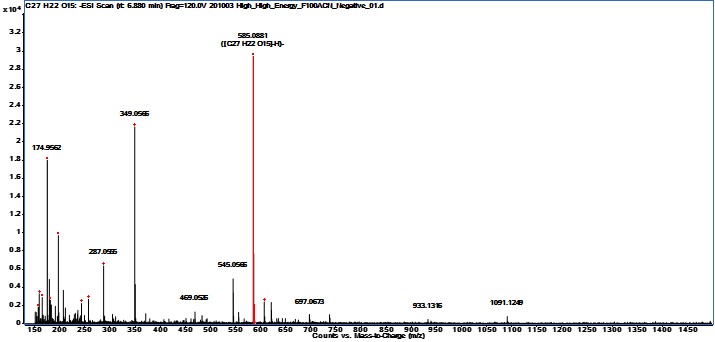


**Figure S42.** MS^1^ spectra data of compound **20** [M-H]^-^=585.0881.


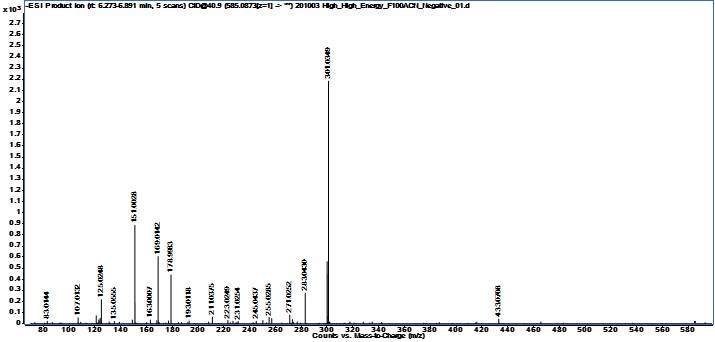


**Figure S43.** MS^2^ spectra data of compound **20** [M-H]^-^=585.0881.


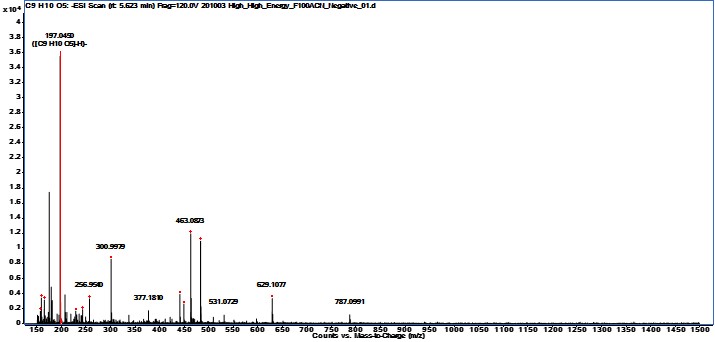


**Figure S44.** MS^1^ spectra data of compound **21** [M-H]^-^=197.0450.


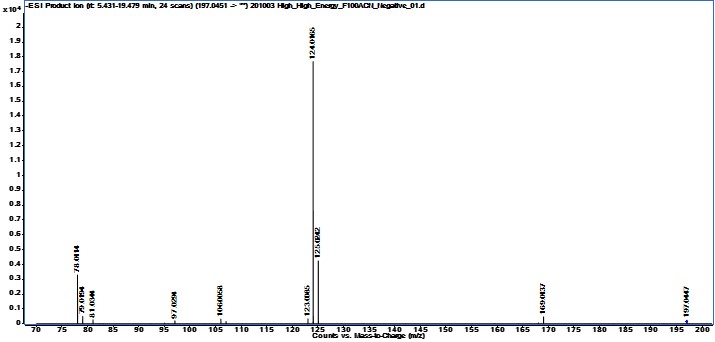


**Figure S45.** MS^2^ spectra data of compound **21** [M-H]^-^=197.0450.


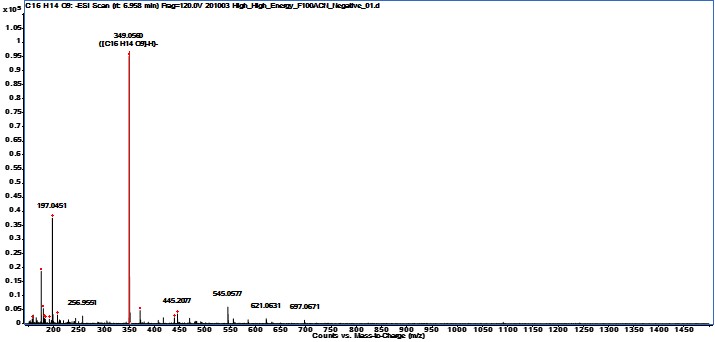


**Figure S46.** MS^1^ spectra data of compound **22** [M-H]^-^=349.0560.


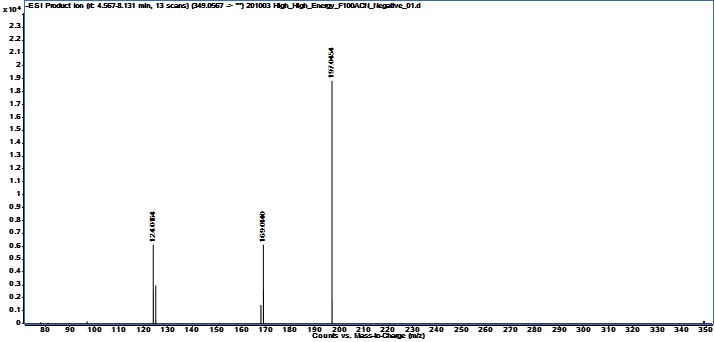


**Figure S47.** MS^2^ spectra data of compound **22** [M-H]^-^=349.0560.


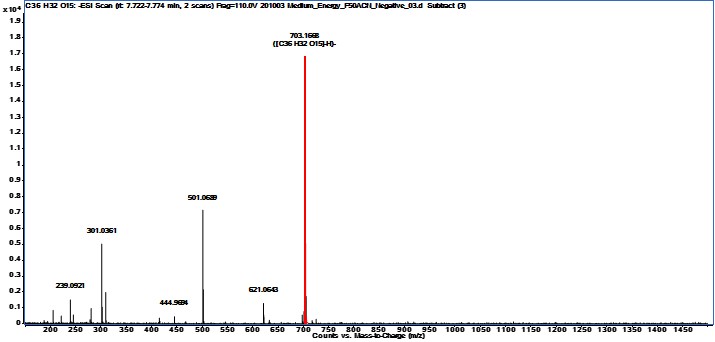


**Figure S48.** MS^1^ spectra data of compound **23** [M-H]^-^=703.1668.


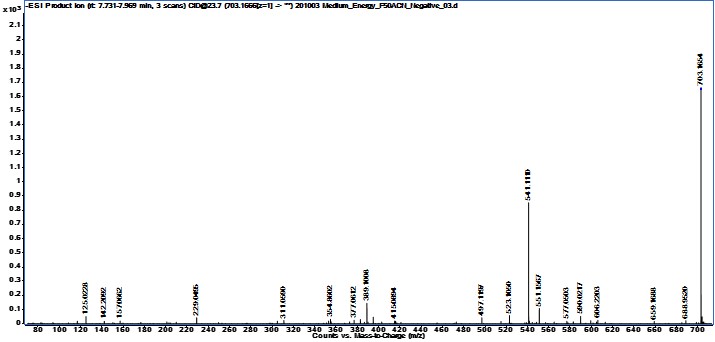


**Figure S49.** MS^2^ spectra data of compound **23** [M-H]^-^=703.1668.


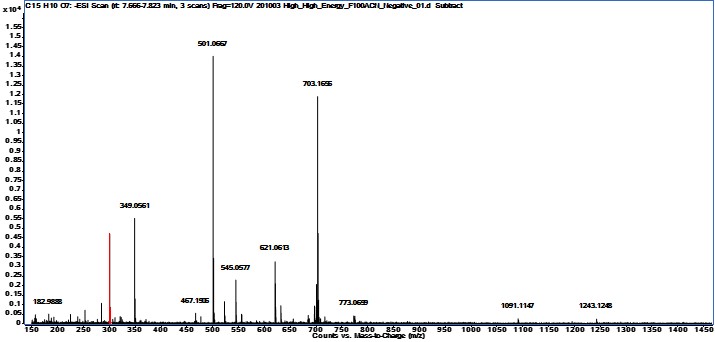


**Figure S50.** MS^1^ spectra data of compound **24** [M-H]^-^=301.0348.


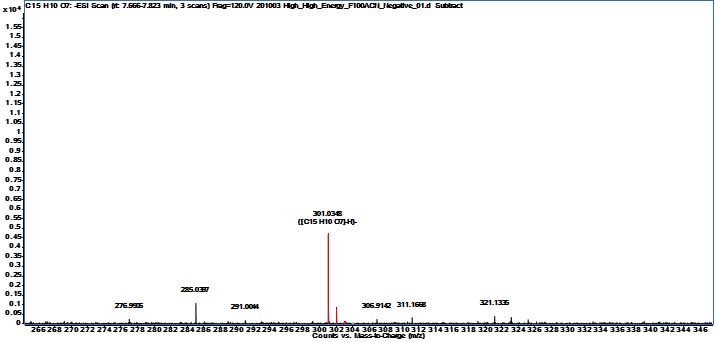


**Figure S51.** Expansion of MS^1^ spectra data of compound **24** [M-H]^-^=301.0348.


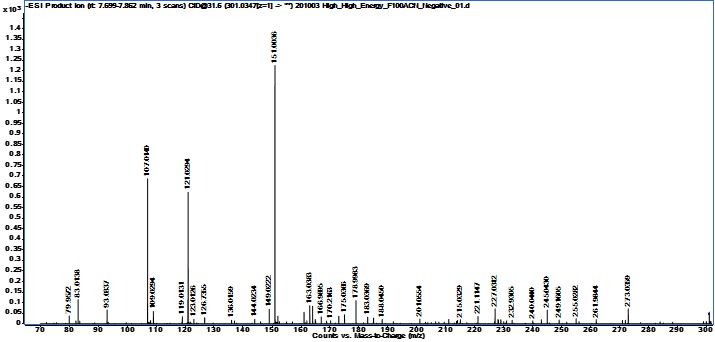


**Figure S52.** MS^2^ spectra data of compound **24** [M-H]^-^=301.0348.


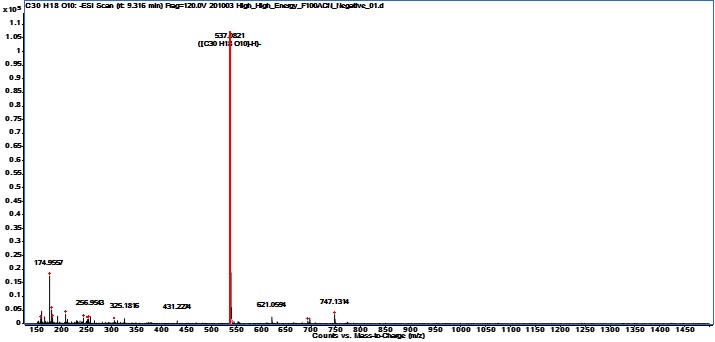


**Figure S53.** MS^1^ spectra data of compound **25** [M-H]^-^=537.0821.


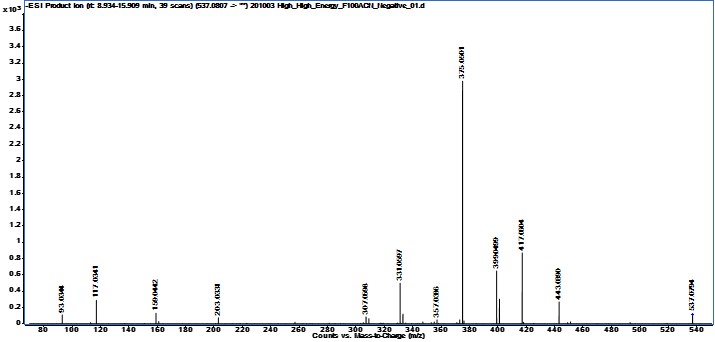


**Figure S54.** MS^2^ spectra data of compound **25** [M-H]^-^=537.0821.


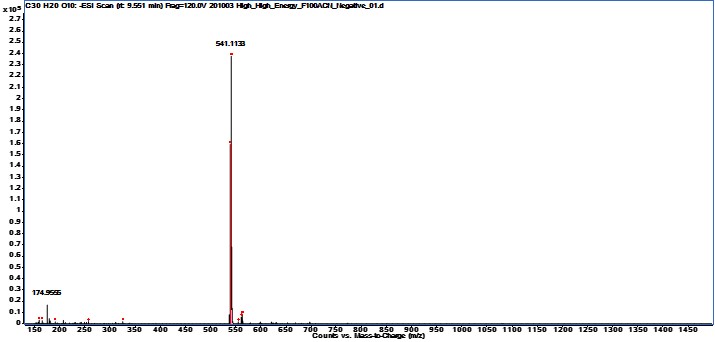


**Figure S55.** MS^1^ spectra data of compound **26** [M-H]^-^=539.0980.


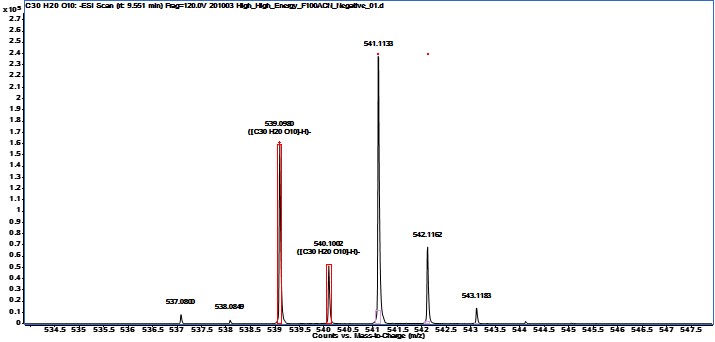


**Figure S56.** Expansion of MS^1^ spectra data of compound **26** [M-H]^-^=539.0980.


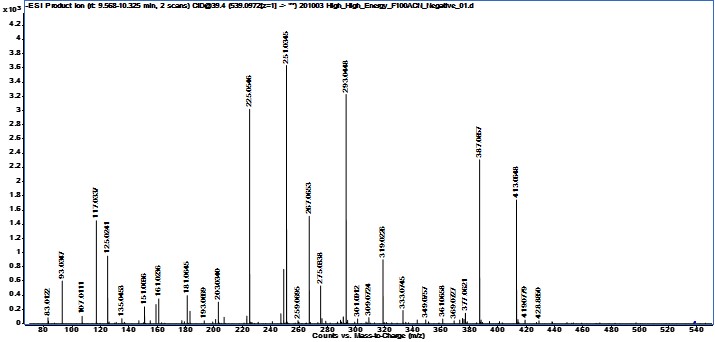


**Figure S57.** MS^2^ spectra data of compound **26** [M-H]^-^=539.0980.


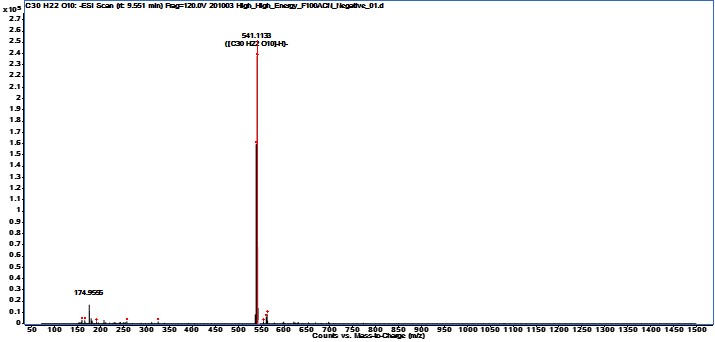


**Figure S58.** MS^1^ spectra data of compound **27** [M-H]^-^=541.1133.


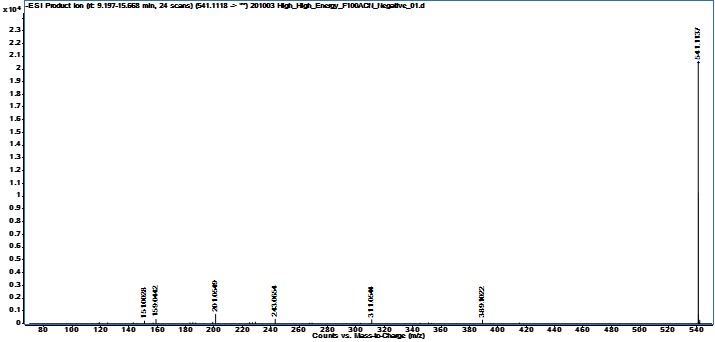


**Figure S59.** MS^2^ spectra data of compound **27** [M-H]^-^=541.1133.


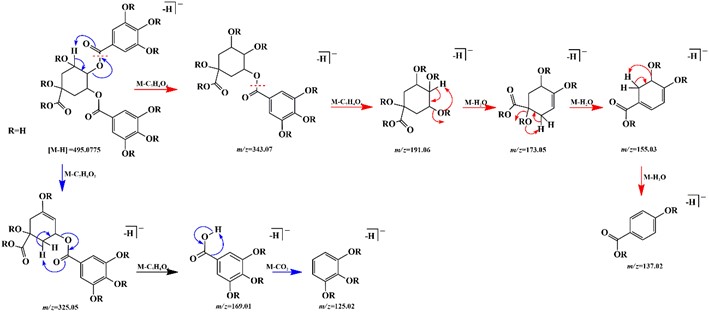


**Figure S60.** Schematic fragmentation pattern of galloyl derivatives obtained from 3,4-di-o-galloylquinic acid.


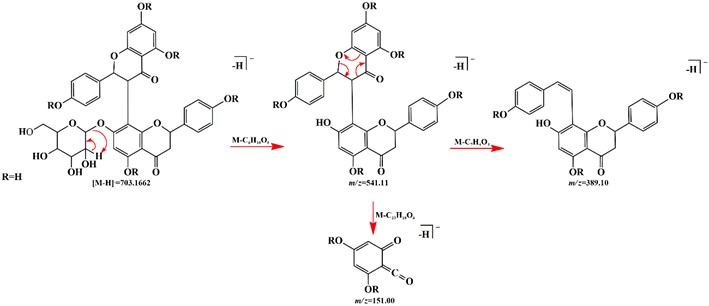


**Figure S61.** Schematic fragmentation pattern of flavonoid obtained from fukugentin-7’’-glucose.


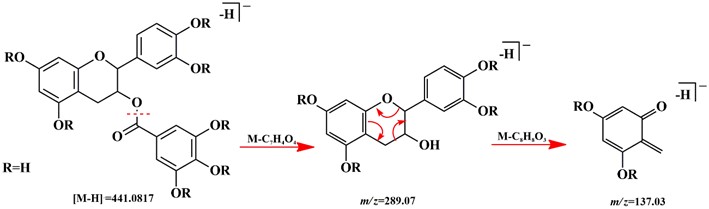


**Figure S62.** Schematic fragmentation pattern of catechin obtained from catechin-3-O-gallate.
